# Review and Future Perspectives of Stimuli-Responsive Bridged Polysilsesquioxanes in Controlled Release Applications

**DOI:** 10.3390/polym16223163

**Published:** 2024-11-13

**Authors:** Xin Zhang, Han Zhang, Xiaonan Liu, Jiao Wang, Shifeng Li, Peng Gao

**Affiliations:** 1 Shandong Key Laboratory of Digital Traditional Chinese Medicine, Institute of Pharmaceutical Research, Shandong University of Traditional Chinese Medicine, Jinan 250355, China; 18304625304@163.com; 2 Department of Pharmacy, Shandong University of Traditional Chinese Medicine, Jinan 250355, China; 60030150@sdutcm.edu.cn (H.Z.); m19861401972@163.com (J.W.); 15552210517@163.com (S.L.)

**Keywords:** stimuli-responsive, bridged polysilsesquioxanes, organosilicon, controlled release, targeted therapeutics

## Abstract

Bridged polysilsesquioxanes (BPSs) are emerging biomaterials composed of synergistic inorganic and organic components. These materials have been investigated as ideal carriers for therapeutic and diagnostic systems for their favorable properties, including excellent biocompatibility, physiological inertia, tunable size and morphology, and their extensive design flexibility of functional organic groups to satisfy diverse application requirements. Stimuli-responsive BPSs can be activated by both endogenous and exogenous stimuli, offering a precise, safe, and effective platform for the controlled release of various targeted therapeutics. This review aims to provide a comprehensive overview of stimuli-responsive BPSs, focusing on their synthetic strategies, biocompatibility, and biodegradability, while critically assessing their capabilities for controlled release in response to specific stimuli. Furthermore, practical suggestions and future perspectives for the design and development of BPSs are presented. This review highlights the significant role of stimuli-responsive BPSs in advancing biomedical research.

## 1. Introduction

Stimuli-responsive delivery systems have been proposed as a means to regulate the release rate of targeted therapeutics, maintain stable blood concentrations, and optimize therapeutic efficacy [1,2]. These systems offer a more precise, safer, and more effective approach to controlled release [3,4]. Despite significant progress in the development of controlled release systems [5,6,7,8], achieving precise therapeutic targeting remains a challenge in clinical research. The efficacy of therapeutics delivered via controllable release systems is influenced not only by the properties of targeted therapeutics and carriers but also by the precision and control of the delivery mechanisms [9,10]. The design of advanced stimuli-responsive carriers aims to enhance the precision, control, and targeting of therapeutic delivery, which has become a primary focus in controlled release system research.

Organosilicon materials are among the leading candidates for constructing stimuli-responsive biomaterials, owing to the biocompatibility of the Si-O-Si bond and the tunability of organic components, which collectively determine their suitability for biomedical applications [11]. Among these, bridged polysilsesquioxanes (BPSs) have attracted considerable attention, owing to their biocompatibility, physiological inertness, and structural tunability [12,13]. As a result, BPS materials are considered promising candidates for the development of stimuli-responsive biomaterials.

While numerous reviews have been published on stimuli-responsive mesoporous silica nanoparticles for drug delivery and cancer therapy [14,15,16,17], there is a notable lack of comprehensive reviews addressing the use of stimuli-responsive BPSs in controlled release systems. This paper seeks to provide a systematic overview of stimuli-responsive BPS biomaterials and their applications in controlled release systems, with a particular focus on their biomedical potential. The review begins with an exploration of design strategies for stimuli-responsive bridged organosiloxanes and the synthesis methods for BPS biomaterials. This is followed by a summary of stimuli-responsive BPS applications in controlled release systems, specifically within biomedical contexts. The biocompatibility and biodegradability of BPS materials are also discussed in detail. Finally, factors that may hinder the clinical development of stimuli-responsive BPSs are considered, along with future perspectives for their application in controlled release systems. This review aims to provide new insights and promising directions for the design and use of stimuli-responsive BPSs in biomedical applications.

## 2. Strategic Design of Stimuli-Responsive BPSs

BPSs are hybrid organic–inorganic materials characterized by their ability to integrate both organic and inorganic components [18]. Their structure consists of [O_1.5_Si-R-SiO_1.5_] units, where R represents the organic bridged group, and the inorganic network is made up of silsesquioxane [SiO_1.5_] [19,20]. The organic bridged groups are covalently bonded to the inorganic silicon atoms via Si-C bonds, resulting in an even distribution of organic components within the silsesquioxane skeleton at the molecular level, as opposed to simple physical mixing [21,22]. The organic bridged groups are highly tunable, allowing for extensive design possibilities to regulate the stimuli-responsive properties of BPSs [23]. This adaptability makes BPS materials highly promising for the development of innovative biomaterials that can enable precision diagnosis and treatment.

### 2.1. Construction of Stimuli-Responsive Bridged Organosiloxanes

**Figure 1:**
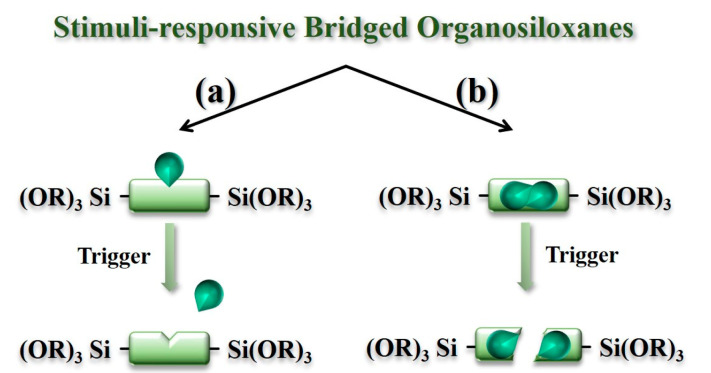
Schematic illustration for different stimuli-responsive bridged organosiloxanes with stimuli-responsive moiety located at side chain (**a**) and main chain (**b**) of organic bridged groups and their degradation processes after trigger treatment.

The performance and functionality of BPSs are determined by the properties of their bridged organosiloxanes, making the design of these monomers crucial for achieving specific material characteristics [24]. The positioning of stimuli-responsive groups within the organic bridged chains is closely linked to the mode of control and release behavior of the stimuli-responsive BPSs. One design strategy involves attaching the stimuli-responsive groups to the side positions of the organic bridged chains (Figure 1a). Upon stimulation, these side groups detach from the main chains, altering the surface properties of the BPS and thus controlling the release of therapeutics adsorbed on the surface. A second approach incorporates the stimuli-responsive groups directly into the main chains of the monomers (Figure 1b). In response to stimuli, the organic bridged chains break apart, causing the disintegration of the BPS and releasing the encapsulated therapeutics.

The flexible designability and broad tunability of the organic bridged groups have made BPS materials a focus of significant research. The synthetic route chosen depends on the characteristics of the bridged organosiloxane monomers and may include organoalkoxysilane functionalization, hydrosilylation, or metallization [19]. Organoalkoxysilane functionalization is a widely used method for monomer synthesis, offering the advantage of starting from readily available raw materials to prepare a wide variety of bridged groups. However, the starting materials must be selected carefully to ensure that each step in the material preparation process adheres to pharmacopoeial standards. In practical applications, the final products must satisfy basic safety and non-toxicity requirements after stimulation. Therefore, strict screening of stimuli-responsive groups and mechanisms is essential. Considerations such as high yield, good biocompatibility, and low cost are critical in the design and development of these materials.

### 2.2. Synthesis of Stimuli-Responsive BPSs

BPS nanoparticles (BPNPs) have garnered significant attention in research, owing to their nanostructure and design flexibility [25]. The surfactant-templated sol–gel method is the most widely employed technique for synthesizing monodisperse BPNPs [26]. This approach utilizes surfactants, such as cetyltrimethylammonium chloride (CTAC) and cetyltrimethylammonium bromide (CTAB), as structural templates, with organic bridged organosiloxanes serving as the sole silicon source to produce periodic mesoporous BPNPs. Studies have shown that the morphology, size, pore size, and structure of BPNPs can be significantly influenced by adjusting reaction parameters such as the type of template agent, the ratio of starting materials, silicon source, pH, reaction temperature, and duration [27]. In addition to periodic mesoporous BPNPs, researchers have focused on mesoporous BPNPs synthesized through the co-condensation of organic bridged siloxanes with tetraethoxysilane (TEOS) or tetramethoxysilane (TMOS) as mixed silicon sources. These particles exhibit larger specific surface areas, allowing for a higher therapeutic loading capacity [28]. However, the reduced organic content in mesoporous BPNPs compared to their periodic counterparts may limit their stimuli responsiveness and degradability in biological environments. The modified Stöber method is frequently employed to synthesize non-porous BPNPs with higher organic content [24,29]. This technique utilizes alcohol as a co-solvent, with ammonia or sodium hydroxide serving as a base catalyst, and relies solely on bridged organosiloxane monomers as the silicon source. The sol–gel process in water produces spherical BPNPs [30]. However, the modified Stöber method cannot achieve precise control over the monodispersity of the BPNPs because of the absence of structure-directing agents.

Shea et al. [31] fabricated a variety of alkylalkene, phenylalkylalkene, and alkylamino BPS nano- or microparticles, systematically controlling the average particle size between 20 nm and ~1.5 μm by adjusting parameters such as 1-propanol content. A research prepared two types of ditelluride-based BPNPs (DTeMSN1 and DTeMSN2) using bis[3-(triethoxysilyl)propyl]ditelluride (BTETePD) as a precursor [32]. BTETePD was added to TEOS to obtain the co-precursors, which were reacted with the structure-directing agents CTAT and triethanolamine to synthesize DTeMSNs via the sol–gel method. Two DTeMSNs with different Te contents were designed by adjusting the mass ratios of TEOS to BTETePD, DTeMSN1 = 4:1, and DTeMSN2 = 3:2, respectively. The BET-specific surface area, total pore volume, and average pore size of DTeMSN2 were 623.4 m^2^ g^−1^, 1.13 cm^3^ g^−1^, and 8.8 nm, respectively, and that of DTeMSN1 were 596.7 m^2^ g^−1^, 1.13 cm^3^ g^−1^, and 7.6 nm, respectively. The results indicate that an increase in ditelluride precursor doping leads to an increase in average pore size.

**Figure 2:**
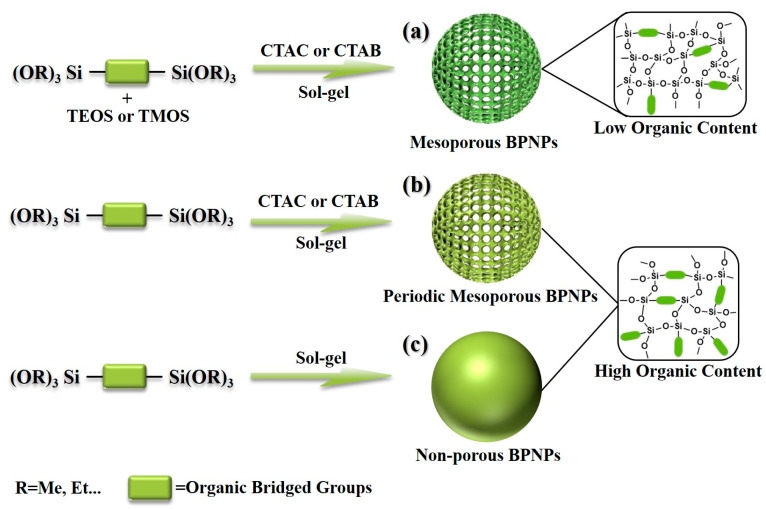
Structures and synthetic pathways of various BPNPs: mesoporous BPNPs (**a**), periodic mesoporous BPNPs (**b**), and non-porous BPNPs (**c**).

Figure 2 illustrates the structure and synthesis pathways of various BPNPs, including mesoporous BPNPs (low organic content), periodic mesoporous BPNPs (high organic content), and non-porous BPNPs (high organic content). Notably, hollow BPNPs have garnered significant attention and are increasingly employed in controlled release systems due to their high specific surface area, tunable porosity, and exceptional loading capacity. Typically, these hollow BPNPs are synthesized using hard template methods, which is an effective approach enabling the stable and controlled hollow structures of BPNPs. For instance, Ha et al. [33] utilized polystyrene beads as a hard template and coated their surface with an organosiloxane layer. Subsequently, monodisperse hollow BPNPs were successfully prepared by selectively etching the polystyrene core through the introduction of ammonia.

The versatility of BPS materials, both chemically and biologically, is expected to play a central role in future biomedical research. The following sections present a comprehensive discussion of stimuli-responsive BPS biomaterials and their potential applications as carriers for the controlled release of targeted therapeutics in biomedical contexts.

## 3. Stimuli-Responsive BPSs in Biomedical Applications

Recent advances in stimuli-responsive BPS biomaterials have attracted significant attention globally, owing to their ability to facilitate the controlled release of targeted therapeutics based on the specific temporal and spatial conditions of various diseases [14,34]. For example, the tumor microenvironment exhibits distinct characteristics compared to normal tissue, such as acidity, hypoxia, elevated enzyme production, and high levels of reactive oxygen species. These conditions can act as physiological signals for environment-specific therapies [35,36,37]. BPS biomaterials offer a versatile platform for developing stimuli-responsive carriers by adapting to different signals from various sources.

**Figure 3:**
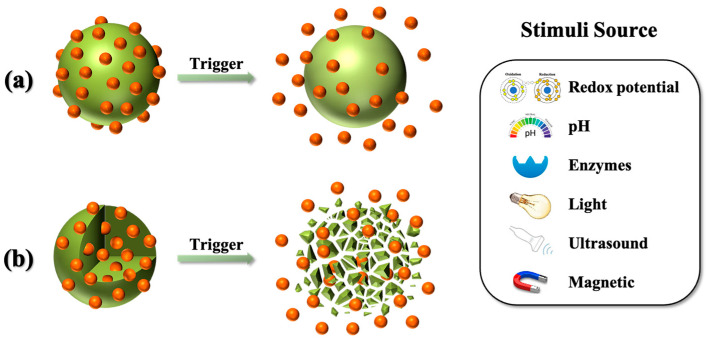
Stimuli-responsive BPS controlled release systems mediated by the triggered degradation of the organic bridged side (**a**) or main chain (**b**).

Targeted therapeutics can be incorporated into BPS materials either through covalent bonds, such as amide or ester linkages, or through non-covalent interactions, including hydrogen bonding, hydrophilic interactions, and electrostatic forces [38]. These therapeutics can either be adsorbed on the BPS’s surface or encapsulated within its structure. Upon stimulation (redox potential, pH, enzymes, light, ultrasound, and magnetic), BPS carriers undergo structural transformation or degradation, resulting in the release of the therapeutics (Figure 3). By adjusting key parameters such as the concentration, duration, and location of the stimuli, the release behavior—encompassing release site, dosage, and timing—can be precisely controlled [39].

This section provides a summary of the strategies used in the development of controlled release systems based on stimuli-responsive BPS biomaterials. These carriers can respond to single or multiple stimuli, and the fundamental mechanisms that govern the release of targeted therapeutics are explored.

### 3.1. Endogenous Stimuli-Responsive BPS Materials

Endogenous stimuli-responsive systems, also known as closed-loop controlled release systems, are triggered by physiological signals. These systems leverage homeostatic regulation in vivo to achieve targeted therapeutic release [40]. In contrast to exogenous stimuli-responsive systems, endogenous systems can more effectively regulate the dosage and rate of therapeutic release based on biological signals related to disease progression, making them particularly suitable for controlled release applications [9,41]. A controlled release system driven by physiological signals comprises a sensor and an actuator, which together facilitate the delivery and release of therapeutics [15,42]. To enhance the release efficiency, biocompatible stimuli-responsive organic bridged groups are selected to induce physical or chemical changes, leading to contraction, rupture, degradation, or alteration of the BPS carrier’s surface properties [13,14,43]. Based on known in vivo physiological stimuli, BPS carriers can be classified into redox-responsive, pH-responsive, and enzyme-responsive materials.

#### 3.1.1. Redox-Responsive

Redox-responsive carriers are primarily activated by intracellular glutathione (GSH) and reactive oxygen species (ROS)-dependent systems [44]. The responses triggered by GSH and ROS not only promote therapeutics’ penetration but also coexist with other stimuli in multi-responsive carriers [45]. Reduction-sensitive carriers rupture in the presence of elevated GSH concentrations, leading to rapid drug release. Similarly, oxidation-responsive carriers are activated by the elevated ROS levels found in pathological conditions such as tumors, atherosclerosis, cardiac and neurological damage, and inflammation. Redox-responsive bridged polysilsesquioxanes (BPSs) incorporate cleavable disulfide, tetrasulfide, diselenide, ditelluride, thioacetal, and thioketal bonds, which break down in response to heightened GSH or ROS levels, enhancing biodegradation and promoting the release of therapeutics [46,47,48]. At this stage, redox-responsive controlled release systems offer optimal efficacy by exploiting intracellular and extracellular redox gradients for the precise delivery and release of therapeutics upon cellular entry. This selectivity contrasts with conventional acid-unstable linkers, which can degrade in low pH environments near tumors, or enzyme-sensitive linkers, which are susceptible to cleavage in the circulatory system. A summary of reported redox-responsive BPS platforms is provided in Table 1.

**Figure 4:**
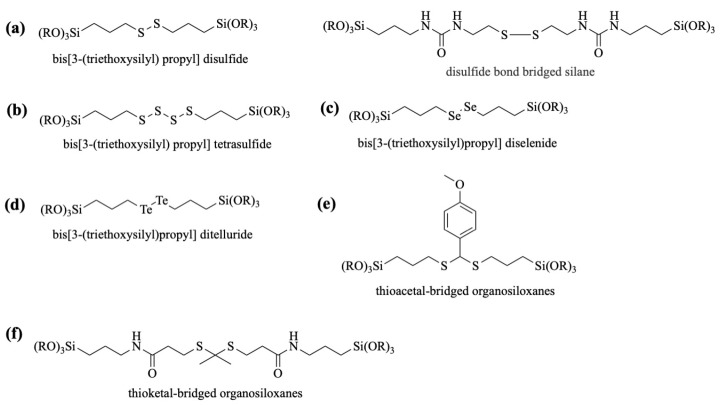
Schematic structure of redox-responsive bridged organosiloxanes containing (**a**) disulfide, (**b**) tetrasulfide, (**c**) diselenide, (**d**) ditelluride, (**e**) thioacetal, and (**f**) thioketal bonds. (R = Me or Et).

Figure 4 illustrates the schematic structure of several redox-responsive bridged organosiloxanes. Disulfide/tetrasulfide bonds are covalently linked to the silicon atom via an inactive group such as an alkyl group or a reactive group such as a ureido group to achieve a reductive response under GSH or reducing agents (Figure 4a,b). The diselenide and ditelluride groups are generally covalently incorporated via propyl groups to two trialkoxysilane to form redox-responsive organic bridging groups, which cleave under either GSH or ROS (Figure 4c,d). Bis[3-(trialkoxysilane) propyl] disulfide, bis[3-(trialkoxysilane) propyl] tetrasulfide, bis[3-(trialkoxysilane)propyl] diselenide, and bis[3-(trialkoxysilane)propyl] ditelluride have been commercially produced for direct purchase and use. The thioacetal- and thioketal-bridged organosiloxanes are produced by chemical synthesis. The thioacetal-bridged organosiloxanes in Figure 4e, for example, are obtained by a nucleophilic reaction of the sulfhydryl group (-SH) in two equivs. of 3-mercaptopropyltriethoxysilane (MPTAS) with the aldehyde group (-CHO) in p-anisaldehyde, thereby siloxylating ROS-responsive thioether bonds. On the other hand, the reaction of 3-aminopropyltrialkoxysilane (APTAS) with structurally symmetric thioketal derivatives causes thioketal-bridged organosiloxanes (Figure 4f), so that both sides of the thioether bond are connected to the two (trialkoxysilane)propyl groups by amide bonds. MPTAS and APTAS are commonly used silane coupling agents in the synthesis of bridged organosiloxane monomers.

Disulfide (S-S) and tetrasulfide (S-S-S-S) compounds exhibit stability under physiological and oxidative conditions, yet they can be reduced to thiols in the presence of a specific quantity of GSH or reducing agents such as dithiothreitol (DTT) [70]. These highly reactive bonds are widely employed in the construction of stimuli-responsive systems that react to reduction. They can serve as linkers or cross-linkers in controlled release systems, facilitating the precise release of targeted therapeutics upon exposure to a reducing environment.

**Figure 5:**
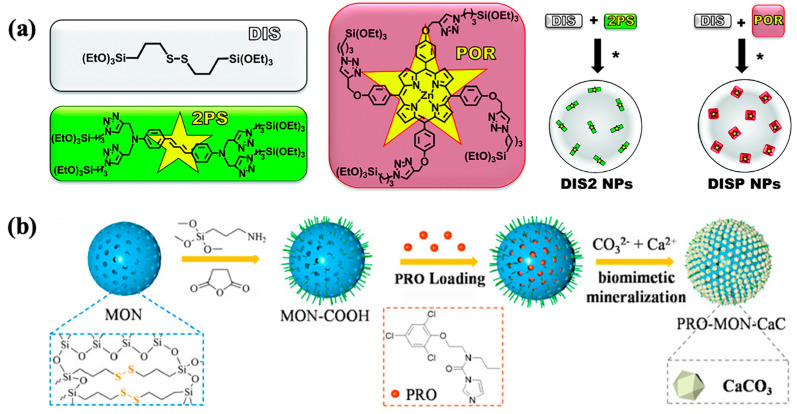
(**a**) Bis(3-triethoxysilylpropyl)disulfide (DIS), two-photon electron donor (2PS), and porphyrin (POR) sol–gel precursors; synthesis of disulfide-based (DIS) and hybrid DIS/2PS (DIS2) or DIS/POR (DISP) BS NPs. Reprinted with permission from [49]. Copyright 2015, The Royal Society of Chemistry. (* represents H_2_O/EtOH, 80 °C/2 h) (**b**) Scheme summarizing preparation of bioresponsive and biodegradable PRO-MON-CaC. Reprinted with permission from [50]. Copyright 2020, American Chemical Society.

**Figure 6:**
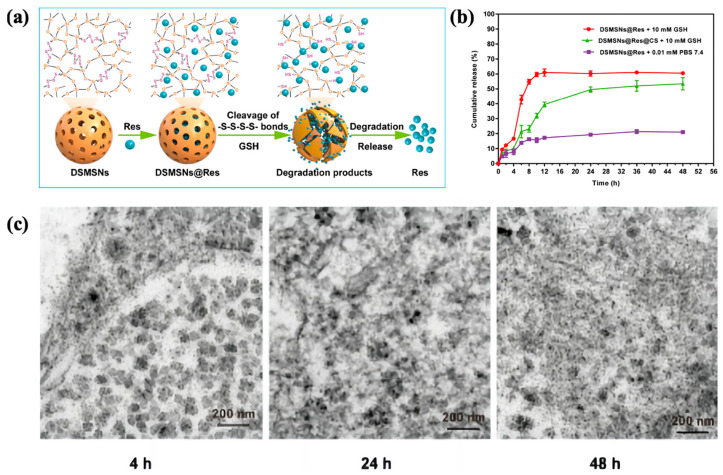
In vitro release and degradation behavior of DSMSNs@Res@CS. (**a**) Schematic illustration of release and degradation mechanism of DSMSNs@Res@CS. (**b**) Cumulative release profiles of Res from DSMSNs@Res@CS in absence and presence of GSH (10 mM). Three experiments were performed with mean ± SD. (**c**) TEM images showing structural evolution of DSMSNs@Res@CS incubated with RAW264.7 cells stimulated by lipopolysaccharide (LPS) for various periods (4, 24, and 48 h). Scale bar = 200 nm. Reprinted with permission from [51]. Copyright 2023, Elsevier B.V.

Croissant et al. [49] reported the development of biodegradable BP nanodevices for fluorescence imaging and the treatment of MCF-7 cancer cells. Two tetra-alkoxysilylated precursors, a two-photon photosensitizer diaminodiphenylbutadiene-bridged siloxane (2PS), and a porphyrin-bridged siloxane photosensitizer (POR) were developed via click chemistry. GSH-responsive nanodevices were synthesized via the co-condensation of disulfide-bridged siloxane (DIS) with 2PS or DIS, resulting in DIS2 (DIS/2PS) and DISP (DIS/POR) BS NPs, respectively (Figure 5a). In near-physiological conditions, these nanodevices demonstrated biodegradability in the presence of GSH. Cellular uptake was confirmed through fluorescence imaging, and spatially targeted cancer cell destruction was achieved via two-photon irradiation. Cao’s research group [50] developed a redox-responsive nanosystem tailored to plant disease microenvironments, employing disulfide-bridged mesoporous organosilicon nanoparticles (MONs) as porous nanocarriers and calcium carbonate (CaC) as a capping agent for prochloraz (PRO) delivery to treat Sclerotinia disease, as shown in Figure 5b. This system, PRO-MON-CaC, exhibited specific responsiveness to disease-associated stimuli, enabling the controlled release of PRO. Biosafety evaluations indicated that the nanocarrier was safe for rapeseed plants at defined doses. In another study, Jiang et al. [51] synthesized tetrasulfide-bridged organosilicon nanoparticles (DSMSNs). After encapsulating resveratrol (Res), the nanoparticles were coated with chondroitin sulfate (CS) for targeted delivery, forming DSMSNs@Res@CS (Figure 6). The CS coating facilitated selective accumulation in colon epithelial cells and macrophages. Upon stimulation by GSH, the redox-responsive switch was activated, triggering the release of Res and subsequent nanoparticle degradation. This strategy enhanced the intracellular delivery of Res in a colitis model. Some of the literature summaries of disulfide/tetrasulfide-based GSH-responsive BPS materials for controlled release applications [49,50,51,52,53,54,55,56,57,58,59,60,61,62] are presented in Table 1.

**Figure 7:**
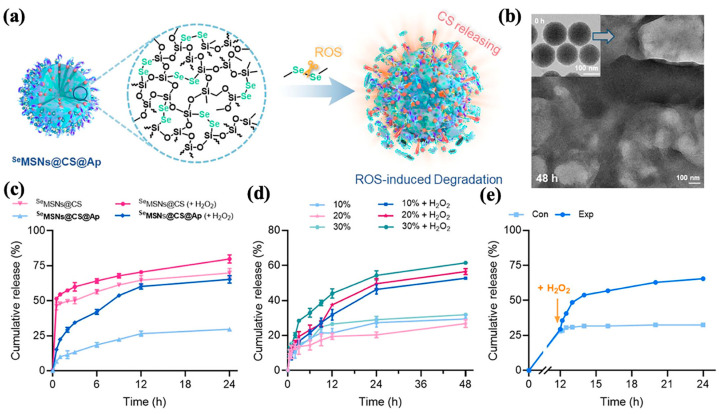
In vitro ROS-responsive biodegradation and drug release behavior of the nanostabilizer. (**a**) A schematic illustration of the ROS-triggered biodegradation of ^Se^MSNs@CS@Ap and the following CS release. (**b**) TEM image of ^Se^MSNs@CS@Ap biodegradation under excessive H_2_O_2_ conditions for 48 h incubation. (**c**) Drug release profiles of ^Se^MSNs@CS and ^Se^MSNs@CS@Ap with/without H_2_O_2_. (**d**) Drug release profiles of ^Se^MSNs@CS@Ap (different selenium wt %) incubated with/without H_2_O_2_. (**e**) Drug release profiles of ^Se^MSNs@CS@Ap incubated at 37 °C for 12 h and then sudden addition of 100 μM H_2_O_2_ for 24 h extension. Con: PBS buffer; Exp: PBS buffer with H_2_O_2_ addition 12 h later. All data are mean ± SD (*n* = 3). Reprinted with permission from [67]. Copyright 2024, American Chemical Society.

The use of tellurium and selenium bonds is gaining recognition as a promising approach for constructing redox-responsive controlled release systems [71]. Xia et al. [32] successfully integrated ditelluride (Te-Te) bonds into mesoporous BPNPs (DTeMSNs), with their surfaces coated in a nanocomplex of poly(ethylene glycol)–curcumin (PEG-CCM). The resulting nanocarrier, DTeMSN@DOX@PEG-CCM, demonstrated sustained DOX release and controlled degradation in response to redox stimuli. This sustained release was attributed to the efficient reaction of redox agents (GSH or H_2_O_2_) with Te-Te bonds in the cancer cell environment, leading to the decomposition of the DTeMSN framework. The addition of PEG-CCM further enhanced cellular uptake and tumor inhibition, while also enabling real-time tracking of cellular uptake, drug release, and biodistribution through a self-fluorescent response. Chen et al. [66] developed MON-Pt@CM nanocarriers incorporating diselenide (Se-Se)-bridged groups for a redox-responsive controlled release of cisplatin in chemotherapy, achieving both efficiency and safety. The diselenide-bridged mesoporous organosilicon nanoparticles (MONs) loaded cisplatin (Pt) by coordination binding between active cisplatin and a selenium atom, resulting in MON-Pt. Then, MON-Pt@CM was constructed via coating MON-Pt with the cancer cell membrane (CM) to achieve a long blood circulation time and high tumor accumulation. A biodegradable hybrid mesoporous organosilicon nanostabilizer, ^Se^MSNs@CS@Ap, was also developed as a potential treatment for allergic diseases [67]. The drug cromoglycate sodium (CS) was loaded into the small pores of diselenide-bridged MSNs (^Se^MSNs) to obtain ^Se^MSNs@CS with a positive charge. Followed by capping with negatively charged IgE aptamer to avoid cargo leakage, we finally provided the corresponding nanocarrier, ^Se^MSNs@CS@Ap. The diselenide-bridged groups within ^Se^MSNs@CS@Ap cleave in response to excessive intracellular ROS, enabling biodegradation and the precise release of the drug CS (Figure 7). A continuous release of CS over 24 h was observed when H_2_O_2_ levels were elevated, addressing the issue of sudden drug release. Additionally, targeted H_2_O_2_ stimulation at specific intervals was shown to accelerate drug release when required.

**Figure 8:**
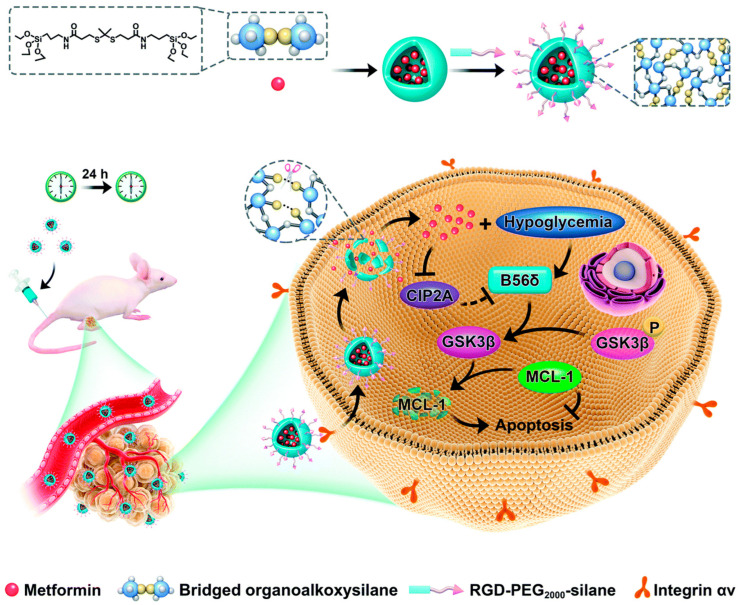
Schematic of ROS-sensitive organosilica nanoparticles for the targeted delivery of metformin against cancer in combination with fasting-induced hypoglycemia. Reprinted with permission from [69]. Copyright 2021, The Royal Society of Chemistry. (B56δ, subunit of protein phosphatase 2A; CIP2A, cancerous inhibitor of protein phosphatase 2A; GSK3β, glycogen synthase kinase 3β; MCL-1, myeloid cell leukemia-1).

Thioacetal and thioketal bonds undergo oxidative cleavage in the presence of ROS, resulting in the formation of thiols and aldehydes or ketones [72]. These bonds are frequently utilized in the construction of ROS-responsive controlled release systems, as the ROS-induced degradation of thioacetal/thioketal groups allows for the on-demand release of therapeutics, improving their delivery efficiency. Lin et al. [68] employed (3-mercaptopropyl) trimethoxysilane and *p*-anisaldehyde in a nucleophilic substitution reaction to synthesize thioacetal-bridged organosiloxanes (BTMPTA), which were then converted into ROS-responsive hollow organosilicon nanoparticles (HMONs) through a hydrolysis–condensation reaction followed by etching. The surface of HMONs was coated with a polydopamine biofilm and aminomethoxy poly(ethylene glycol) to prevent premature DOX leakage, resulting in the controlled release system DOX@HMONs@PDA-mPEG. This system exhibited structural collapse upon ROS exposure, triggered by the cleavage of thioacetal bonds. Yu et al. [69] demonstrated the ROS-responsive degradable bridged silsesquioxane nanoparticles (BS-NPs) using thioketal (TK)-bridged organoalkoxysilanes as precursors. The TK-bridged BS-NPs with a uniform size of 50 nm could encapsulate drug metformin to form BS-NPs@M. A targeting peptide RGD-PEG_2000_-silane was conjugated to the surface of BS-NPs@M to gain tumor-targeted BS-NPs (T-BS-NPs@M), facilitating efficient delivery to cancer cells (Figure 8). Because of the ROS-sensitive matrix containing thioketal bonds, metformin was specifically released from the nanocarriers in a controlled manner within tumor cells.

Redox-responsive BPS carriers display excellent biocompatibility and effectively penetrate cancer cells to deliver therapeutics in a controlled manner, attracting considerable research interest. However, the specific release mechanisms of these carriers within pathological microenvironments may influence cellular redox levels, and the underlying processes require further clarification. Additionally, the metabolic pathways of post-response degradation products from redox-responsive BPS carriers remain unclear and warrant detailed investigation. While redox-responsive BPS systems exhibit significant potential for on-demand therapeutics release, their clinical application is considerably challenging. Addressing these issues necessitates collaborative efforts focused on carrier development, toxicological evaluation, and pharmacokinetic studies within an industrial context.

#### 3.1.2. pH-Responsive

pH-responsive controlled release systems function in response to changes in acidic and alkaline environments [73]. Low pH values are characteristic of human skin, wound sites, cancerous tissues, and other physiological environments [74,75,76]. Leveraging these pH differences, carriers are designed to release therapeutics in specific pH conditions, enabling extended circulation times, reduced premature leakage, and targeted molecular release. This strategy enhances the therapeutic efficacy of targeted treatments while minimizing side effects [77,78].

pH-responsive release is primarily achieved through structural changes in the organic bridged groups of BPS carriers, which respond to different pH levels [79,80]. These materials are widely applied in the delivery and controlled release of cancer therapies, vaccines, insulin, and pesticides. The release mechanisms can be categorized as follows: (1) Specific chemical bonds in the carrier break because of pH-induced changes in bond stability. For example, acid-sensitive substances, such as protic acid esters, hydrazones, and amides, contain acid-cleavable bonds that enable pH-responsive release [81]. (2) The variation in pH disrupts hydrogen bonds formed between specific groups in the BPS carriers and the therapeutics, causing the controlled release of therapeutics from the carriers [82]. (3) The protonation and deprotonation balance of BPS carriers shifts with pH changes, altering the surface charge properties of the system. This affects the electrostatic interactions between the carriers and the therapeutics, facilitating pH-responsive release [83]. Common pH-sensitive groups include carboxyl, amino, and pyridine groups. For instance, carriers containing basic groups (-NH_2_, -NHR, -NR_2_) or acidic groups (-COOH) undergo deprotonation and protonation, respectively. At high pH levels, -COOH groups deprotonate, immobilizing or encapsulating the therapeutics via electrostatic attraction, while at low pH levels, these groups protonate to form -NH_3_⁺, causing electrostatic repulsion and the subsequent release of the therapeutic. Additionally, the release profile can be further modulated by adjusting the number of pH-responsive groups. A summary of reported pH-responsive BPS platforms is provided in Table 2.

**Figure 9:**
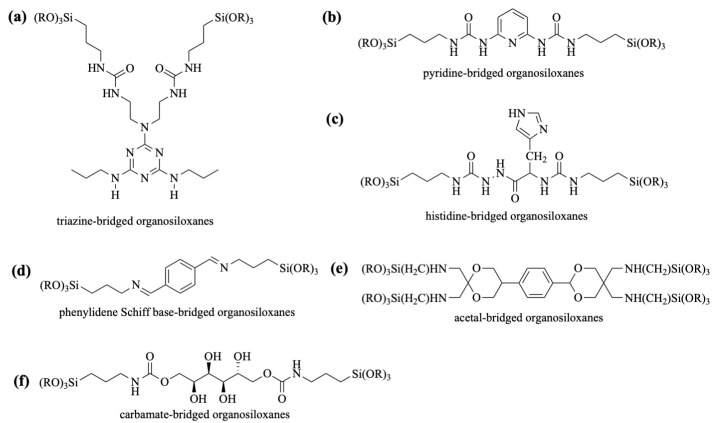
Schematic structure of pH-responsive bridged organosiloxanes. (**a**) Triazine-bridged organosiloxanes, (**b**) pyridine-bridged organosiloxanes, (**c**) histidine-bridged organosiloxanes, (**d**) phenylidene Schiff base-bridged organosiloxanes, (**e**) acetal-bridged organosiloxanes, and (**f**) carbamate-bridged organosiloxanes (R = Me or Et).

The pH-responsive bridged organosiloxane monomers are mostly organically bridged with amino, urea, amide, acetal, and carbamate groups in their structures, as shown in Figure 9. In the structures of triazine-, pyridine-, and histidine-bridged organosiloxanes (Figure 9a–c), all of them contain urea groups (-NH-CO-NH-). Through this group, the pH-responsive derivative is covalently linked to trialkoxysilane. This reaction is generally made by a nucleophilic reaction of -NCO in 3-isocyanatopropyltrialkoxysilane with -NH_2_ in the pH-responsive derivative. Similarly, the carbamete group (-NH-COO-) in carbamete-bridged organosiloxanes (Figure 9f) is produced by reaction of -NCO with the hydroxyl group in sorbitol. The introduction of a Schiff base has been prepared by reacting 3-aminopropyltrialkoxysilane with dialdehydes (Figure 9d). Alkylhalide-substituted acetal derivatives have been used with -NH_2_ of 3-aminopropyltrialkoxysilane to give acetal-bridged groups with amino bonds being covalently linked to four trialkylsiloxanes (Figure 9e). These pH-responsive groups or derivatives have symmetrical structures containing reactive groups on both sides, facilitating the reaction with silane coupling agents to build bridging groups.

**Figure 10:**
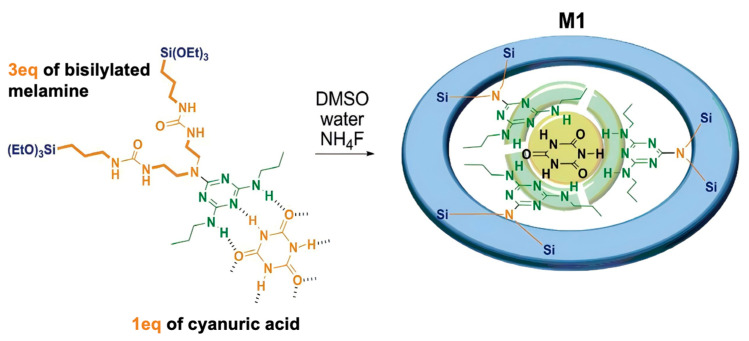
Preparation of M1. Reprinted with permission from [84]. Copyright 2011, American Chemical Society.

The hydrogen atom in the amino group and the oxygen atom in the carbonyl group form stable hydrogen bonds under neutral conditions, but these bonds break in acidic environments. Leveraging this principle, pH-responsive controlled release systems have been developed. Carcel et al. [84] synthesized tetrasilylated precursors with triazine derivatives as organic bridged groups, creating imprinted pH-responsive BPS materials (M1) with fiber-like structures via a sol–gel process (Figure 10). These materials exhibit dual functionality, with molecular recognition and targeted release. The study employed molecular imprinting methods via hydrogen bonding interactions to construct bridged siloxane capable of releasing imprinted molecules under mildly acidic conditions. Bis(silylated) triazine derivatives were bonded to cyanuric acid via hydrogen bonds. The potential for cyanuric acid release from this pH-responsive BPS carrier was examined at 37 °C under strongly acidic and moderate pH conditions. The results confirmed that cyanuric acid could be effectively released in mildly acidic media (pH = 5.5) without compromising the integrity of the BPS material. Similarly, non-porous BPS nanomaterials with triazine bridges were developed by interacting 5-fluorouracil (5-FU) through weak hydrogen bonds, creating a pH-sensitive BPS drug delivery system (BS) [85]. Carcel and collaborators [86] introduced fluorescein isothiocyanate onto a triazine-derivative BPS carrier, resulting in a novel non-porous BPS system (nano-BS) that cleaves hydrogen bonds under acidic conditions, releasing cyanuric acid. This system is applicable for both targeted release and fluorescent cell imaging.

**Figure 11:**
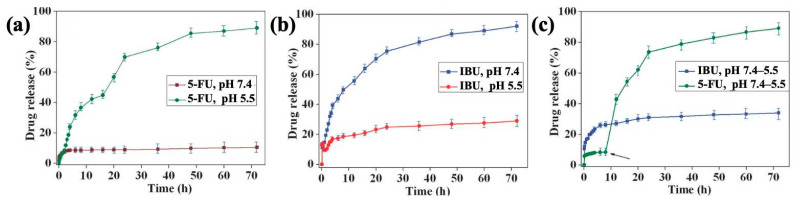
Time-dependent release kinetics of (**a**) 5-FU and (**b**) IBU from the HMCs-20 in a PBS buffer at pH 7.4 and 5.5 at 37 °C. (**c**) The percentage release of the drugs from the dual-drug delivery system according to the pH of the release medium. The arrow indicates the point at which pH 7.4 was changed to pH 5.5. Reprinted with permission from [87]. Copyright 2013, Wiley-VCH Verlag GmbH & Co. KGaA, Weinheim, Germany.

Moorthy et al. [87] synthesized mesoporous organosilica hybrid microcarriers (HMCs) containing diurea and pyridine functionalities for pH-triggered drug release. The hydrophilic anticancer agent 5-FU and the hydrophobic nonsteroidal anti-inflammatory drug ibuprofen (IBU) were encapsulated in the HMCs through multiple hydrogen bonds and electrostatic interactions and released at pH 5.5 and 7.4, respectively (Figure 11). These HMCs offer a novel approach for dual-drug loading, enabling the precisely controlled release of targeted therapeutics via distinct interaction mechanisms triggered by pH changes. Wang et al. [88] prepared a pH-responsive periodic mesoporous organosilicon material (His-PMO) by condensing histidine-bridged organosiloxane precursors (His-BOP) with tetraethoxysilane (TEOS). Paclitaxel (PTX) was encapsulated in His-PMO through electrostatic interactions, and the pH-triggered controlled release process was observed in vitro. The release behavior of PTX from His-PMO exhibited a distinct pH-responsive mechanism, wherein the imidazole group within the channels was protonated, causing an electrostatic repulsion of PTX under acidic conditions. This shift in electrostatic equilibrium at different pH values altered both the dosage and release rate of the drug.

**Figure 12:**
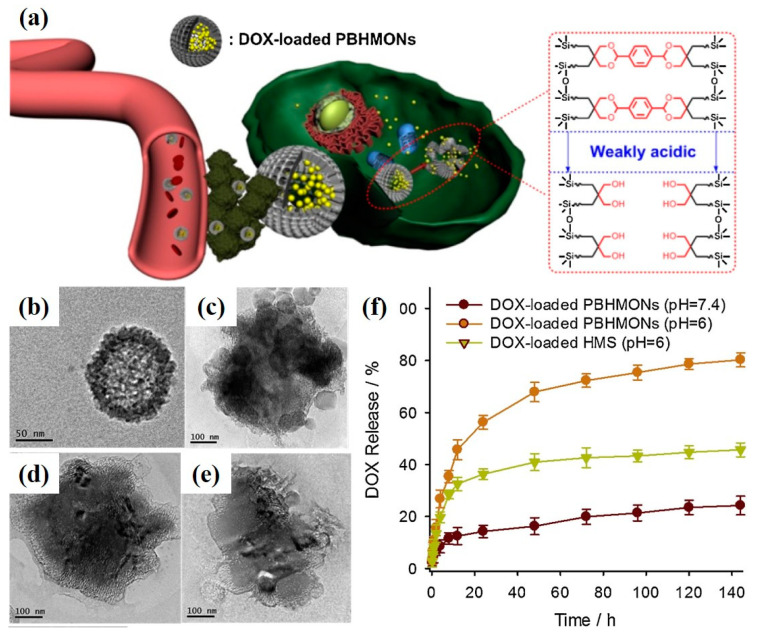
(**a**) Schematic representation of blood transport/tumor accumulation of PBHMONs and their pH-responsive biodegradation. (**b**–**e**) TEM analysis of a suspension of PBHMONs (0.1 mg/mL, PBS, pH = 6, 37 °C) after 0, 0.5, 1, and 3 d. (**f**) DOX release profiles of DOX-loaded PBHMONs at pH = 6.0 and 7.4 and DOX-loaded HMS at pH = 6. Error bars indicate mean ± SD, *n* = 3. Reprinted with permission from [91]. Copyright 2019, Elsevier B.V. (HMS, hollow mesoporous silica).

Yuan et al. [89] employed Schiff base interactions to chemically incorporate phenylidene Schiff bases into bridged MON carriers, yielding S-MON. This novel nanoplatform demonstrated effective pH-triggered degradability, enhancing the release kinetics of targeted therapeutics in acidic environments. Curcho’s group [90] described the development of hybrid nanoparticles (PS@SiO_2_*), utilizing a polystyrene (PS) core coated with an organosilicon framework (SiO_2_). The core–shell pH-responsive coating was constructed using tetraethoxysilane (TEOS) and pH-sensitive diamine-bridged organosiloxanes via a sol–gel process. The study explored the degradation behavior in response to pH changes. Under acidic conditions (pH 5.0), the acid-sensitive organosilicon framework underwent hydrolysis of diimine bonds, leading to rapid degradation of the coating and exposure of the PS nanoparticles. This design of pH-responsive organosilicon coatings offers a simple yet powerful approach for a variety of biological applications, including on-demand therapeutic release, sensing technologies, and smart protective coatings with rapid response times. Yang [91] reported the development of novel hollow mesoporous BPS nanoparticles (PBHMONs) with pH-responsive biodegradability for the controlled release of anticancer drugs (Figure 12). The acetal-bridged moiety within the PBHMONs cleaved in response to weak acids. Doxorubicin (DOX), an anticancer drug, was efficiently loaded into the PBHMONs through π-π interactions with the pH-responsive bridged groups. These PBHMONs demonstrated selective biodegradation in the weakly acidic tumor microenvironment, leading to partial drug release while facilitating rapid clearance, thereby minimizing long-term toxicity. Zharov and colleagues [92] developed water-degradable bridged organosilica nanoparticles (ICPTES-sorbitol SNPs) via the co-condensation of sorbitol-bridged siloxanes containing carbamate linkages with TEOS. Exposure of ICPTES–sorbitol SNPs to aqueous environments at neutral or acidic pH resulted in a gradual degradation from non-porous to porous, culminating in complete breakdown due to the hydrolysis of carbamate bonds.

Research on pH-responsive controlled release systems has gained significant attention. BPS biomaterials with pH sensitivity are increasingly being recognized as promising carriers for the efficient loading and controlled release of therapeutic molecules, including drugs, nucleic acids, and proteins. The construction of these carriers relies on the selection of pH-sensitive organic bridged groups and the mode of binding with therapeutic agents. Variations in pH induce structural modifications in the BPS carriers, including disintegration, changes in surface charge, alterations in hydrogen bonding, and shifts in hydrophilic/hydrophobic properties. These pH-responsive systems can protect therapeutic agents from bioenvironment-induced degradation or loss of efficacy, enhancing bioavailability and enabling sustained release.

#### 3.1.3. Enzyme-Responsive

Enzymes, as key metabolic catalysts, participate in nearly all biological processes [93]. Leveraging enzyme specificity and efficiency for the targeted release of therapeutics offers an effective approach for constructing enzyme-responsive BPS controlled release systems [94]. By utilizing enzyme-responsive bridged groups as substrates, changes in enzyme expression levels trigger modifications in the chemical structure of the substrates, leading to the disintegration of the carrier and controlled release of the encapsulated therapeutics. A summary of reported enzyme-responsive BPS platforms is provided in Table 3.

**Figure 13:**
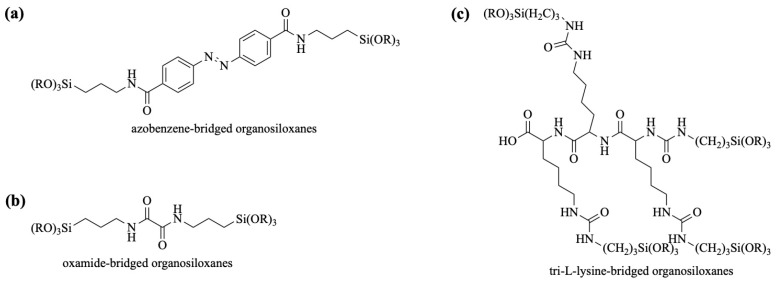
Schematic structure of enzyme-responsive bridged organosiloxanes. (**a**) Azobenzene-bridged organosiloxanes, (**b**) oxamide-bridged organosiloxanes, and (**c**) tri-L-lysine-bridged organosiloxanes (R = Me or Et).

Followed by the substitution of dichloride-functionalized azobenzene with 3-aminopropyltrialkoxysilane, successful silylation of the azobenzene moiety is achieved through two amide bonds (Figure 13a). Similarly, the nucleophilic substitution of oxalyl chloride with two equivalents of 3-aminopropyltrialkoxysilane yields organosiloxanes featuring dual amide bonds as organic bridges (Figure 13b). The reaction between isocyanates and amines to form urea bonds has been previously mentioned. Consequently, four equivalents of 3-isocyanatopropyltrialkoxysilane were employed to react with tri-l-lysine containing four primary amino groups, resulting in the tri-l-lysine-bridged organosiloxanes (Figure 13c).

**Figure 14:**
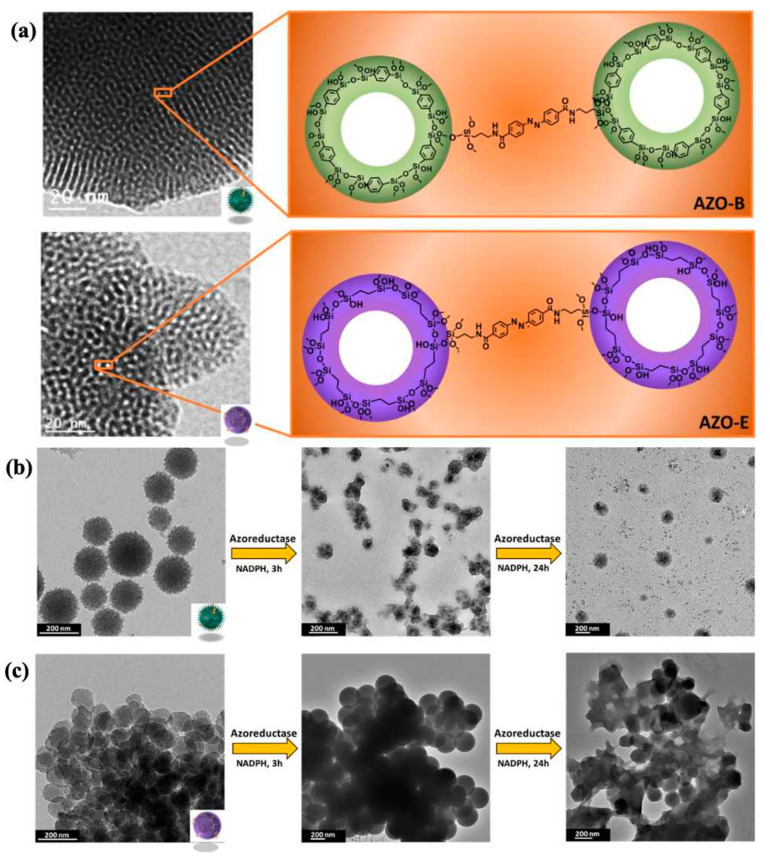
(**a**) Transmission electron microscopy (TEM) and schematic illustration of AZO-B and AZO-E pore walls. TEM images of (**b**) AZO-B and (**c**) AZO-E before and after degradation in azoreductase enzyme in presence of NADPH for 3 and 24 h. Reprinted with permission from [96]. Copyright 2018, American Chemical Society.

Azo bonds serve as particularly useful enzyme-degradable segments in controlled release systems, undergoing reductive cleavage catalyzed by azoreductase enzymes [100]. Li and Tang [95] developed a BPS system containing azo-bridged groups as enzymatically responsive gates for MSNs, where ibuprofen was encapsulated and released upon cleavage of the azo bonds by azoreductase. In another study, Omar et al. [96] co-condensed azobenzene-bridged siloxanes (AZOs) with aromatic benzene (B)- and aliphatic ethane (E)-bridged siloxanes, resulting in the formation of AZO-B and AZO-E, respectively (Figure 14a). AZO-B and AZO-E enzymatically degraded in an azoreductase enzyme in the presence of nicotinamide adenine dinucleotide phosphate (NADPH), as displayed in Figure 14b,c. The results showed that they provided a compact pore–wall framework and enhanced enzyme-responsive biodegradation catalyzed by azoreductase, enabling the controlled release of therapeutics.

**Figure 15:**
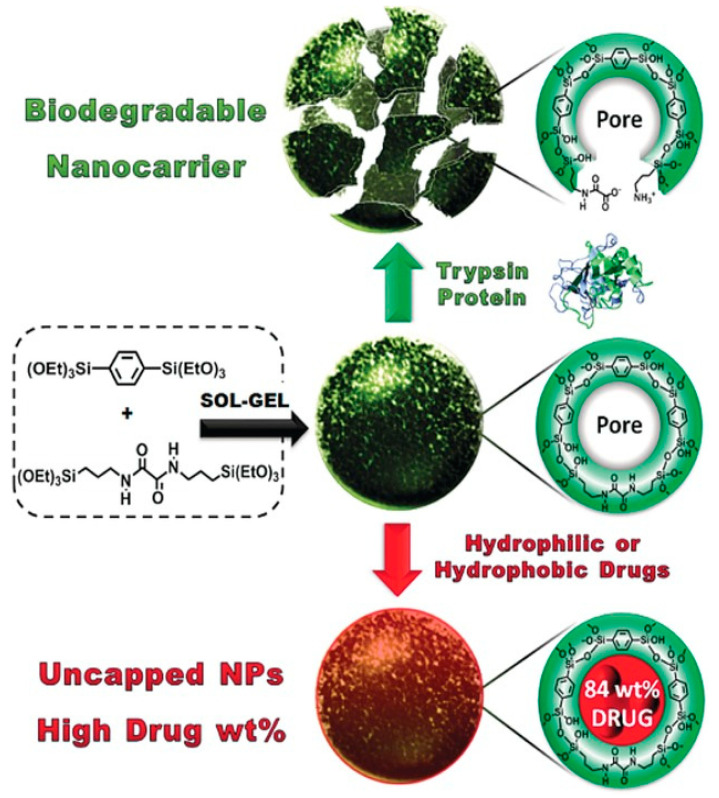
Representation of the sol–gel synthesis of MONs, their pore structure before and after protein-mediated degradation (**top**) or after high drug loadings, affording nonleaky NPs with uncapped pores (**bottom**). Reprinted with permission from [98]. Copyright 2016, Wiley-VCH Verlag GmbH & Co. KGaA, Weinheim, Germany.

Khashab and colleagues [97] designed biodegradable bridged silsesquioxane nanofluorescent probes (BS NPs) with high organic content, introducing fluorescein isothiocyanate into enzyme-responsive Si-O-Si frameworks to enhance cancer cell imaging. These nanoprobes degraded in response to trypsin, breaking the oxamide-bridged groups. Additionally, the group synthesized biodegradable oxamide phenylene-based mesoporous organosilicon nanoparticles (MONs) by co-condensing oxamide-bridged siloxanes with phenylene-bridged siloxanes [98]. These nanoparticles exhibited high organic content and drug-loading capacity, facilitating controlled release in cancer cells triggered by a trypsin model protein through enzyme-responsive degradation (Figure 15). Maggini et al. [99] synthesized tri-l-lysine (Lys_3_)-bridged BPS nanodonuts (NDs), which possessed approximately 70% organic content, enhancing cellular uptake. The enzymatic degradation of these nanodonuts led to controlled therapeutic release.

Thus, enzyme-responsive systems offer a direct and stimuli-responsive controlled release strategy by inducing a partial cleavage of bridged bonds within the BPS carrier, resulting in its disassembly upon enzymatic stimulation.

### 3.2. Exogenous Stimuli-Responsive BPSs

Unlike endogenous stimuli, exogenous stimuli provide precise spatial and temporal control, allowing for the targeted delivery of therapeutics in a specific and dose-dependent manner [101]. The responsiveness of BPS carrier systems to exogenous stimuli is determined by the physicochemical properties of the materials, enabling controlled release applications. Upon exposure to exogenous factors, the organic bridged components in BPS carriers undergo cleavage reactions, altering intermolecular interactions and resulting in structural degradation of the carriers. This process facilitates targeted delivery and improves therapeutic efficacy. Common exogenous stimuli include light, magnetic fields, and ultrasound. BPS biomaterials responsive to these stimuli are categorized into light-responsive, magnetic-responsive, and ultrasound-responsive controlled release systems. A summary of reported exogenous stimuli-responsive BPS platforms is provided in Table 4.

#### 3.2.1. UV Light

Light, as a clean, efficient, and environmentally friendly energy source, exhibits unique properties such as remote regulation and rapid responsiveness [114]. Additionally, it can be used in a non-contact manner to regulate materials with precision in terms of timing, localization, and quantity [115]. Chemical reactions in light-responsive materials typically occur without the need for additional substances like initiators and are generally unaffected by the surrounding environment, providing favorable conditions for modulating material responses. The rational selection of photoresponsive groups and the design of molecular structures allow for polymeric materials to exhibit one or more light-responsive behaviors, such as photomodulated charge transformation, phototropic deformation, and phototriggered degradation [116]. These properties give photoresponsive controlled release systems high controllability and spatiotemporal resolution, improving drug efficacy and reducing off-target release. Photocleavage groups, which undergo irreversible photolysis upon light irradiation, serve as the primary photoresponsive components [117]. The structure and function of photoresponsive polymer materials can be controlled by selecting appropriate photoresponsive groups and designing molecular structures, for example, optoelectric charge transformation, light-induced deformation, and light-triggered degradation [118]. These groups can be covalently linked to molecules of interest. For instance, when photofracture groups are introduced into the main chain of BPS organic bridges, the entire BPS material undergoes photodegradation upon light stimulation. Alternatively, photofracture groups may act as side-chain groups of the organic bridges, where active groups such as carboxyl, hydroxyl, or amine groups are deprotected under light, altering the surface charge of the BPS material. Reversible photoreaction groups can also be incorporated into the organic bridges to modify the hydrophilicity of the polymer in response to light.

**Figure 16:**
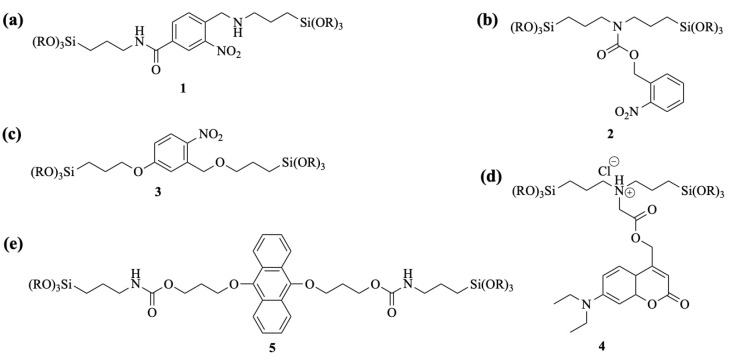
Schematic structure of photoresponsive bridged organosiloxanes. (**a**) *o*-Nitrobenzyl amino-bridged organosiloxanes, (**b**) *o*-nitrobenzyl bis-trimethoxysilylpropyl carbamate, (**c**) *o*-nitrobenzyl ester-bridged organosiloxanes, (**d**) coumarin-bridged organosiloxanes, and (**e**) 9, 10-dialkoxyanthracene-bridged organosiloxanes (R = Me or Et).

The incorporation of photoresponsive *o*-nitrobenzyl groups into organosiloxane structures as organic bridges is commonly achieved by chlorinating the *o*-nitrobenzyl group followed by its reaction with an amino-containing siloxane. For example, the reaction with 3-aminopropyltrialkoxysilane gives compound **1** (Figure 16a), wherein the incorporation of the *o*-nitrobenzyl group into the organic bridged main chain occurs. The *o*-nitrobenzyl group in compound **1**, obtained by a reaction with bis(trialkoxysilylpropyl)amine, exists as an organic bridged side group (Figure 16b). Compound **3** is afforded by the hydrosilylation of bisallylated *o*-nitrobenzyl derivatives with trialkoxysilane (Figure 16c). A simple quaternization reaction is employed to introduce the coumarin derivative into the side group of bis(trialkoxysilylpropyl)amine (Figure 16d). The photocleavable dialkoxyanthracene is covalently linked to two propyltrialkoxysilyl via amide bonds (Figure 16e).

Fatieiev et al. [102] successfully synthesized photoresponsive bridged silsesquioxane nanoparticles (BS NPs) containing a high organic functional component (50%), uniformly distributed throughout the nanomaterial. Upon irradiation with 365 nm light, the organic bridging groups in these nanoparticles underwent photoreaction, resulting in surface charge reversal, which facilitated the photocontrolled transport of plasmid DNA in human cervical cancer cells.

**Figure 17:**
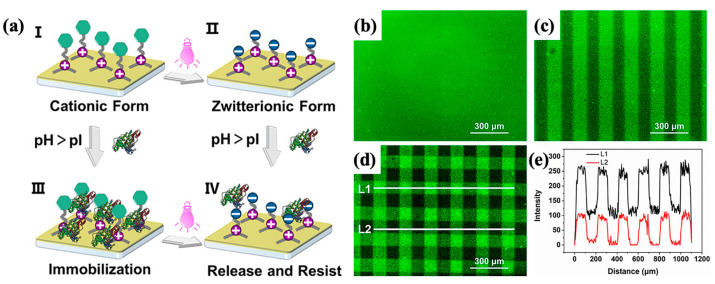
(**a**) Schematic illustration of protein immobilization and release on light-triggered charge shifting polysilsesquioxane surface. Fluorescence microscopy images (**b**–**d**) of sequential release of proteins under temporal and spatial control by light and fluorescence intensity profiles (**e**) along lines in image (**d**). Reprinted with permission from [103]. Copyright 2021, American Chemical Society.

Using a similar approach, Zhu’s group designed and synthesized a series of bridged siloxanes with photoresponsive properties, developing BPS materials that allowed for precise control over the release of targeted therapeutics and the surface micropatterning of bioactive molecules [103,104,105]. By introducing diethylaminocoumarin as the photofunctional unit into the side chain of organic bridges (Figure 17), they created a photoreponsive bridged polysilsesquioxane surface (CBPS) that underwent surface charge alterations under 410 nm light irradiation, enabling the light-controlled fixation and release of proteins to form precise protein micropatterns [103]. By adjusting the irradiation parameters temporally and spatially, the concentration, position, shape, and size of the protein micropatterns can be precisely controlled. Additionally, by incorporating a universal photocleavage group, the *o*-nitrobenzyl moiety, into the organic bridge side chain, Zhu’s team [104,105] synthesized bridged polysilsesquioxane nanoparticles (NBPS) with light-triggered charge reversal properties. By modulating irradiation intensity, duration, and on/off modes, the position, timing, and quantity of therapeutic release can be precisely regulated across multiple dimensions. This class of materials and techniques holds potential for applications in the controlled delivery of food additives, dyes, cosmetics, insecticides, UVA absorbers, and pharmaceuticals.

**Figure 18:**
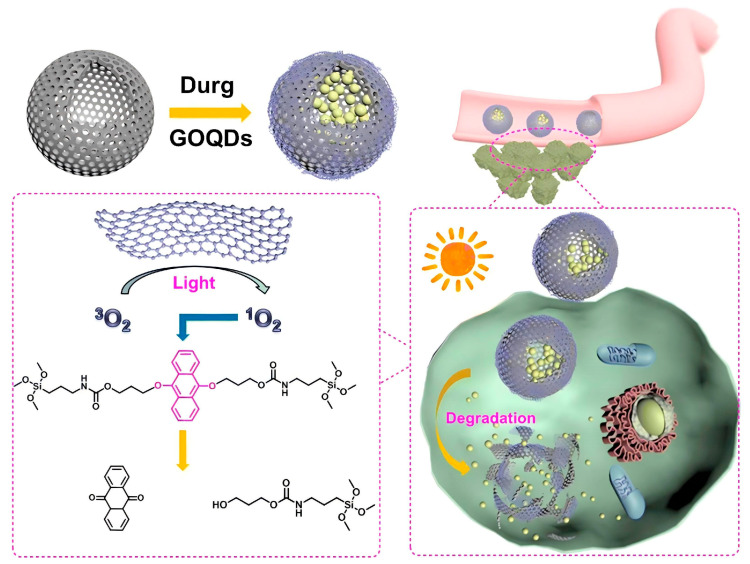
Schematic illustration of organosilica nanoplatform (HMONs@GOQDs) for cancer therapy. Reprinted with permission from [106]. Copyright 2020, the author(s).

Yang et al. [106] synthesized a photoresponsive degradable organosilica nanoplatform (HMONs@GOQD) based on hollow mesoporous organosilicon nanoparticles (HMONs) with a light-responsive degradation mechanism using a 9, 10-dialkoxyanthracene (DN)-bridged alkoxysilane precursor sensitive to singlet oxygen (^1^O_2_). These nanoparticles encapsulated graphene oxide quantum dots (GOQDs) and the drug in the presence of a surfactant and triethylamine. Upon exposure to light, the quantum dots generated ^1^O_2_, leading to nanoparticle degradation and enhanced drug release (Figure 18). A novel photodegradable bis-alkoxysilane containing *o*-nitrobenzyl ether was also synthesized for the preparation of light-breakable organo-bridged mesoporous silica particles (LB-MSPs) [107]. These LB-MSPs, which function as multi-compartment containers for photostimulated cargo release, demonstrated the release of 7-dehydrocholesterol upon ultraviolet irradiation.

Given the simplicity and controllability of exogenous stimuli, extensive research has focused on utilizing photoresponsive BPSs in biomedical applications. Exogenous stimuli allow for precise temporal and spatial control, enabling the development of novel therapeutic methods such as photothermal therapy (PTT), photodynamic therapy (PDT), and synergistic immunotherapy [119]. However, further investigation is needed to ensure the safe dosage of exogenous stimuli. Additionally, exploring whether the degradation products of BPSs under exogenous stimuli could exhibit diagnostic or therapeutic properties would further enhance their potential efficacy. Thus far, preliminary studies have integrated drug research with degradable silicon dioxide nanoparticles.

#### 3.2.2. NIR Light

**Figure 19:**
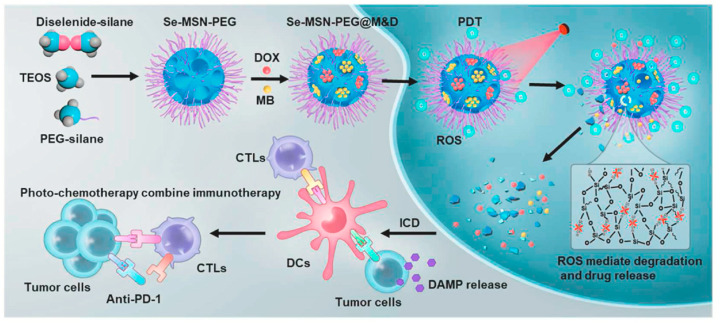
A schematic of the synthetic procedure of Se-MSN-PEG with cascading drug release and amplifying ICD manners and their application for efficient and safe cancer chemo-photoimmunotherapy. Reprinted with permission from [108]. Copyright 2022, Elsevier B.V.

UV light, despite its strong tissue penetration capability, causes significant tissue damage because of its short wavelength, limiting its suitability for clinical applications. Near-infrared (NIR) light, on the other hand, offers several advantages in biological applications, including deep tissue penetration, inherent fluorescence, minimal tissue scattering, and high biocompatibility [120]. Integrating NIR responsiveness with organosilicon materials has led to the creation of intelligent “nanodevices” for the controlled delivery of targeted therapeutics, contributing to significant advancements [121]. Yang et al. [108] utilized polyethylene glycol (PEG)-modified diselenide-based bridged mesoporous organosilicon nanoparticles (Se-MSN-PEG) as carriers for red light-triggered self-destruction. These carriers co-encapsulated the anticancer drug DOX and the photosensitizer methylene blue (MB) to form Se-MSN-PEG@M&D, enabling chemo-photodynamic therapy. During photodynamic therapy, ROS generated by low-dose red light irradiation mediated the cleavage of diselenide-bridged bonds, leading to the degradation of the organosilicon carrier and subsequent drug release (Figure 19). This synergistic property of chemo-photodynamic therapy functions as a cascade amplifier to improve the immunogenic cell death (ICD) effect both in vivo and in vitro. Similarly, Peng et al. [109] developed a light-driven nanotransformer, ID@M-N, using diselenide-bridged bonds along with a thermosensitive outer shell composed of an N-isopropylacrylamide (NIPAM) layer hybridized with indocyanine green (ICG). Under NIR light irradiation, ROS generated by ICG broke down the diselenide bonds, resulting in the rapid degradation of the BPS matrix and the production of smaller fragments containing DOX. This NIR-induced drug release facilitated deep tumor penetration and improved chemotherapy efficacy.

**Figure 20:**
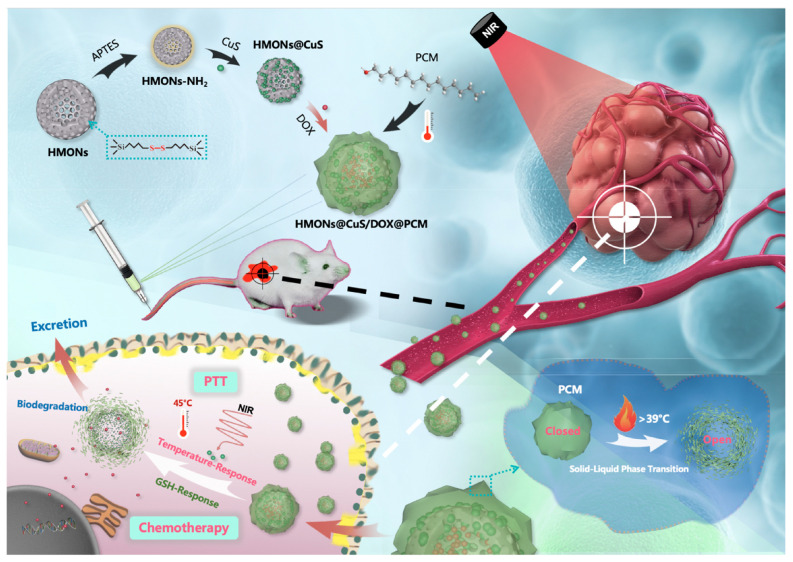
A schematic of the synthesis of HMONs@CuS/DOX@PCM nanosystems with dual-stimuli-responsive drug release for synergistic chemo- and photothermal tumor therapy. Reprinted with permission from [110]. Copyright 2020, Elsevier B.V.

Chen et al. [110] constructed redox/NIR-responsive controlled release nanosystems (HMONs@CuS/DOX@PCM) based on biodegradable hollow mesoporous organosilicon nanoparticles, whose surfaces were functionalized with a phase-change material (PCM) and copper sulfide (CuS) nanocrystals acting as photothermal-responsive “gatekeepers” (Figure 20). The surface of amino-functionalized disulfide-hybridized HMONs were decorated by CuS nanocrystals to synthesize HMONs@CuS nanoparticles. HMONs@CuS/DOX@PCM was constructed, following a capping step for DOX-loaded HMONs@CuS with PCM. The NIR-to-thermal conversion of CuS nanocrystals triggered temperature-responsive behavior in the PCM on the surface of HMONs, enabling the NIR-responsive release of targeted therapeutics. Additionally, the biodegradable HMONs, which contain bisulfide bonds, facilitated GSH-responsive DOX release within tumor microenvironments, achieving a synergistic effect between chemotherapy and photothermal therapy. A similar dual-stimuli-responsive drug delivery system (Dox@CuS-BMSN-HA) was developed using CuS and tetrasulfide for chemo-photothermal synergistic therapy [111]. A mesoporous-structured CuS nanocomposite with a tetrasulfide bond (CuS-BMSN) was employed to load the anticancer drug, resulting in Dox@CuS-BMSN. Hyaluronic acid (HA) was complexed by electrostatic interactions with Dox@CuS-BMSN to gain Dox@CuS-BMSN-HA, achieving multiple functions for drug leakage prevention, enzyme-triggered drug release, and targeting moiety toward CD44-overexpressing cancer cells.

#### 3.2.3. Magnetic

**Figure 21:**
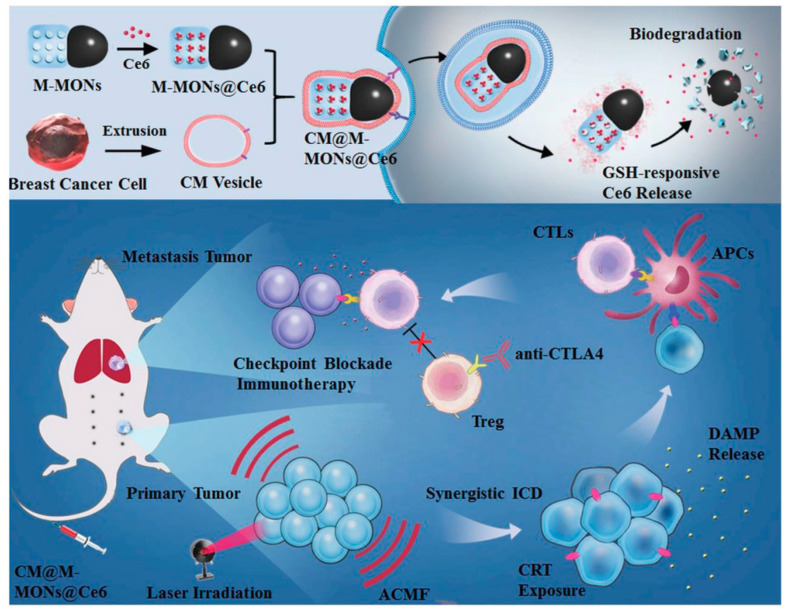
A schematic of the synthetic procedure for the cancer cell membrane-cloaked Ce6-loaded Janus magnetic mesoporous organosilica nanoparticles (CM@M-MONs@Ce6) and their application for combined PDT and magnetic hyperthermia to further potentiate a CTLA-4 blockade to enhance synergistic antitumor immunity in combating cancer metastasis. Reprinted with permission from [112]. Copyright 2019, the authors.

The magnetic-responsive drug delivery method offers a non-invasive approach for precise temporal and spatial control in targeted therapies [122]. In magnetic hyperthermia therapy for tumors, magnetic particles localized at the tumor site are heated to 43–47 °C by an alternating magnetic field, effectively destroying tumor cells [123]. This technology presents several advantages, including targeted delivery, remote control capability, simplified administration, reduced dosage, minimal side effects, and seamless integration with other therapeutic modalities [124]. As a result, it is considered a superior approach for thermal cancer therapy compared to other stimulus-responsive systems, owing to its short response time. The development of a Janus magnetic mesoporous organosilica nanoparticle (M-MON) platform for breast cancer treatment integrates photodynamic therapy (PDT), magnetic hyperthermia, and tumor-specific immunotherapy [112]. M-MONs demonstrate a high loading capacity for the photosensitizer chlorin e6 (Ce6), M-MONs@Ce6, commonly employed in PDT (Figure 21). The asymmetric growth of disulfide-bridged mesoporous organosilica rods on Fe_3_O_4_ nanospheres enables dual reduction and pH-responsive drug release while mitigating long-term in vivo toxicity associated with matrix degradation. The resulting M-MONs@Ce6 nanoparticles are coated with breast cancer cell membranes (CMs) to form CM@M-MONs@Ce6, which enhances accumulation at the tumor site and prolongs blood circulation. This system combines PDT and magnetic hyperthermia to produce potent synergistic anticancer effects, inducing immunogenic cell death and eliciting a tumor-specific immune response.

#### 3.2.4. Ultrasound

**Figure 22:**
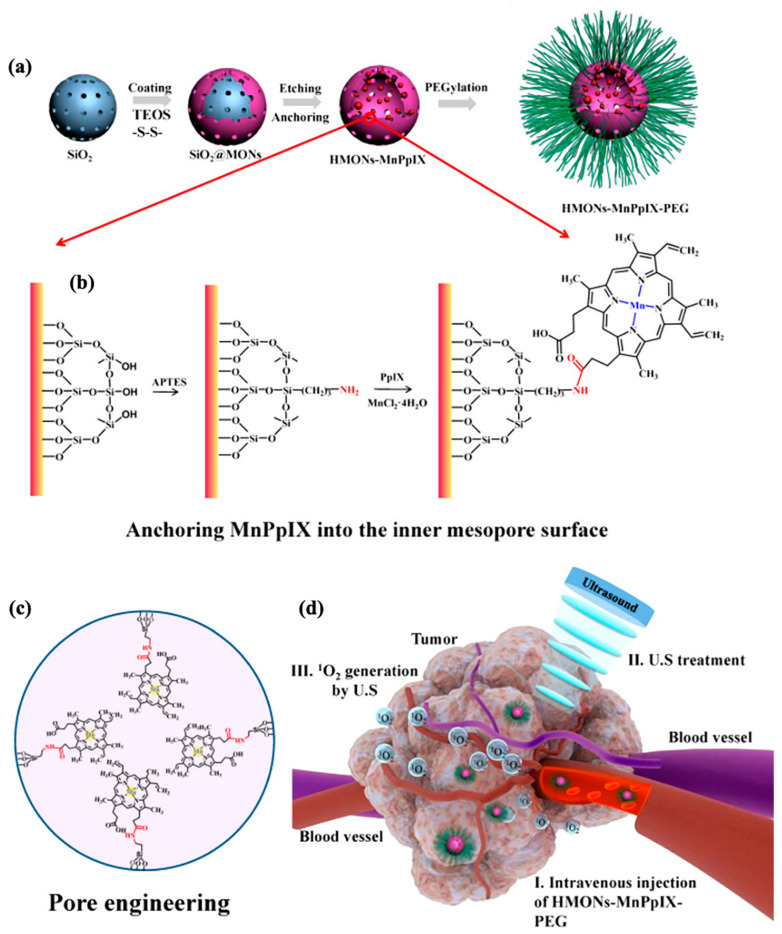
A schematic illustration of (**a**) the synthesis of HMONs-MnPpIX-PEG, (**b**) detailed synthetic steps of grafting MnPpIX into the inner mesopore surface of HMONs to fabricate HMONs-MnPpIX-PEG based on metalloporphyrin chemistry, (**c**) the scheme of MnPpIX covalently grafted into the mesopores, and (**d**) the blood transport/tumor accumulation of HMONs-MnPpIX-PEG and its SDT effect on cancer treatment. Reprinted with permission from [113]. Copyright 2017, American Chemical Society.

Ultrasound (US) is a safe, non-invasive, and radiation-free external stimulation method widely applied in biomedical research [125]. Advances in US-triggered cancer therapy have led to the development of novel nanomaterials, with increasing research on emerging modalities such as high-intensity focused ultrasound (HIFU), sonodynamic therapy (SDT), and US-mediated drug delivery [126]. SDT, an emerging tumor treatment, uses US to activate specific compounds (sonosensitizers) within tumor cells to generate ROS for tumor eradication. Compared to light-based therapies, US offers greater tissue penetration and shows significant potential in cancer treatment [127]. Disulfide bonds (S-S) are covalently cross-linked into the framework of hollow mesoporous organosilica nanoparticles (HMONs) to facilitate mild reductive degradation within the tumor microenvironment [113]. The hollow structure of HMONs is further functionalized by anchoring Mn-protoporphyrin (MnPpIX), forming composite sonosensitizer HMONs-MnPpIX (Figure 22). Stability under physiological conditions is enhanced by PEGylating the surface with methoxy-terminated PEG silanes, resulting in HMONs-MnPpIX-PEG. During SDT, the sonosensitizers, when exposed to US waves, generate ROS that diffuse through the mesopores, enhancing biodegradability and biocompatibility while effectively killing tumor cells. This SDT strategy combines the advantages of ultrasound-based biomedical treatments with nanomedicine, opening new pathways for efficient cancer therapies.

Synergistic therapy leverages multiple treatment methods to exploit the complementary effects of different approaches, enhancing efficacy while reducing side effects associated with individual treatments [128]. BPSs possess unique structural and functional characteristics, making them ideal candidates for constructing synergistic therapeutic nanoplatforms. The functional modification of BPSs with specific components can confer stimulus responsiveness to the carriers, and their high specific surface area and tunable pore structure enable efficient loading of therapeutic agents such as chemotherapy drugs, photosensitizers, sonosensitizers, and magnetic nanoparticles. These properties facilitate the precise control of therapeutic agent release at lesion sites, offering more effective and targeted treatment strategies with broad clinical potential.

### 3.3. Multiple-Stimuli-Responsive BPSs

Recent advancements in dual- and multiple-stimuli-responsive biomaterials have enabled the precise control of release mechanisms [129,130]. Given the complex internal environment of living organisms and the unpredictable progression of diseases, a single-stimuli-responsive biomaterial delivery system often fails to meet therapeutic requirements [131,132]. As a result, dual- or multiple-stimuli-responsive systems are being explored, not only to enhance material properties but also to better address the uncertainties associated with the changing conditions within an organism. Selecting appropriate stimuli-responsive carriers for controlled release allows for more precise treatment tailored to distinct pathological environments. In contrast to single-stimuli-responsive carriers, dual- or multiple-stimuli-responsive systems can react to two or more environmental triggers, making them more suitable for the diverse demands of targeted molecule controlled release [133]. Despite these advantages, clinical applications remain challenging. From a biomaterial perspective, the design and development of multiple-stimuli-responsive carriers is essential to expanding the use of these materials in biomedical applications. A summary of reported multiple-stimuli-responsive BPS platforms is provided in Table 5.

**Figure 23:**
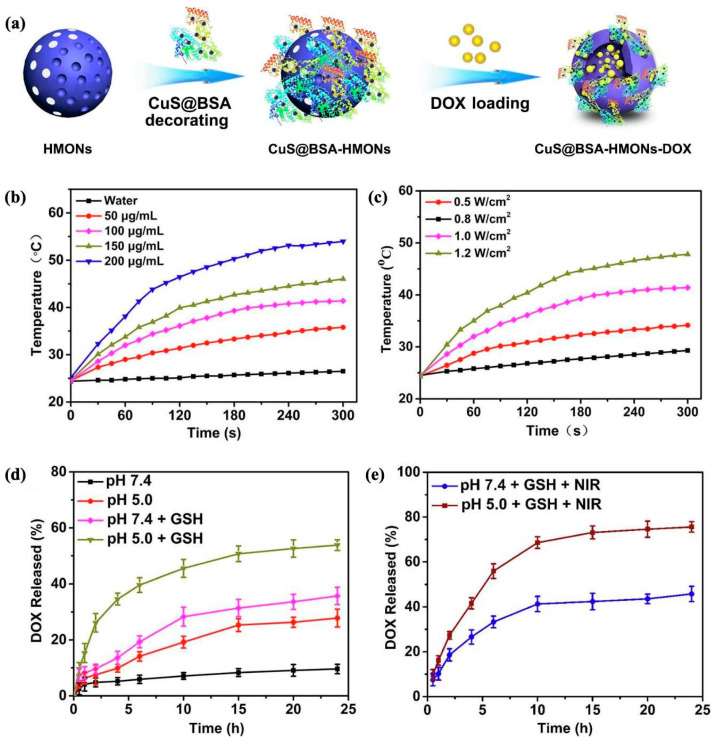
(**a**) A schematic illustration of the synthesis of CuS@BSA-HMONs-DOX nanoparticles. (**b**) Concentration-dependent photothermal curves of CuS@BSA-HMONs aqueous solutions (808 nm laser, 1.0 W cm^−2^). (**c**) Laser power-dependent photothermal curves of CuS@BSA-HMONs aqueous solutions (808 nm laser, 100 µg mL^−1^). (**d**) In vitro DOX release profiles from CuS@BSA-HMONs-DOX at different pH values with or without adding 10 mM GSH. (**e**) In vitro DOX release profiles from CuS@BSA-HMONs-DOX at different pHs in the presence of 10 mM GSH with 808 nm NIR laser irradiation (1 W cm^−2^). Reprinted with permission from [135]. Copyright 2020, Elsevier B.V.

Tang and colleagues [134] synthesized hollow mesoporous organosilica nanoparticles (HMONs) through the co-hydrolysis and co-condensation of bis(triethoxysilyl)phenylene (BTEB) and bis[3-(triethoxysilyl)propyl]tetrasulfide (BTES). The surface of HMONs was further modified by PEG-silane and MPTES to glycolyze and sulfide the surface, anchoring polyoxometalate (POM) through strong Mo-thiol ligand interactions, yielding HMOPM. In acidic tumor environments, these HMOPMs spontaneously self-assemble, enhancing tumor accumulation while also exhibiting excellent GSH-responsive photothermal conversion and photoacoustic imaging capabilities via the reduction of Mo(VI) to Mo(V). This pH/GSH dual-stimuli-responsive organosilicon nanoplatform is promising for tumor-specific self-assembly and synergistic cancer therapy. Li et al. [135] developed a theranostic nanoplatform, CuS@BSA-HMONs-DOX, which demonstrates pH-, GSH-, and NIR-responsive controlled release of DOX (Figure 23a). Their approach used biocompatible nanocomposites (CuS@BSA) as photothermal converters and disulfide-bridged polysilsesquioxanes (HMONs) as biodegradable carriers, achieving synergistic chemo-photothermal therapy in the tumor microenvironment. The mild hyperthermia was precisely controlled by modulating nanoparticles’ concentration (Figure 23b) and the power density and irradiation time of the NIR laser (Figure 23c). The pH/GSH/NIR tri-stimuli-responsive performance of CuS@BSA-HMONs-DOX nanoparticles determined the DOX release amount (Figure 17d,e). Similar studies have been conducted by other research institutions [136,137,138].

**Figure 24:**
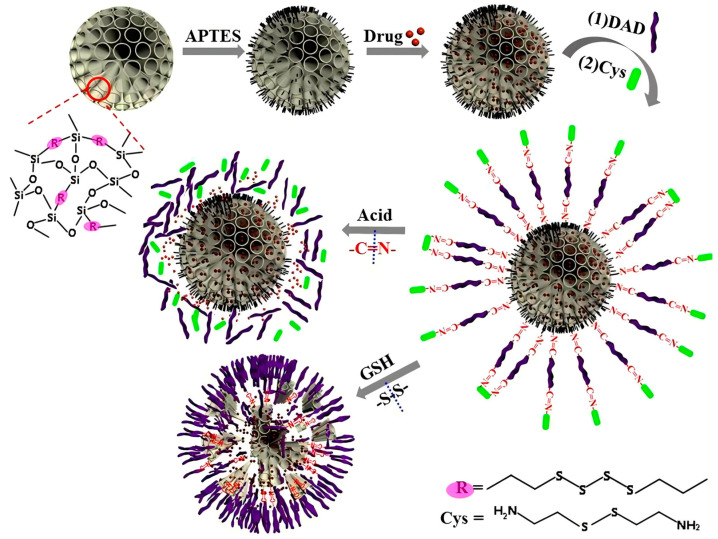
Illustration of preparation, release, and degradation of DOX-loaded OS-N=C-DAD/Cys system. Reprinted with permission from [140]. Copyright 2020, Elsevier B.V.

Ren et al. [139] developed dual pH- and redox-responsive nanocarriers composed of a metal–organic framework (ZIF-8) core and an organosilicon shell containing disulfide bridges. These nanocarriers, termed ZDOS NPs, exhibited a high loading capacity for DOX and achieved dual-stimuli-responsive drug release at low pH and high GSH concentrations. In another study by Li et al. [140], a controlled release system (OS-N=C-DAD/Cys) was constructed using disulfide-bridged organosilicon (OS)-based nanocarriers. These were further functionalized by modifying the surface with dialdehyde dextrin (DAD) through Schiff base bonding (-N=C-) (Figure 24). Cystamine (Cys) was then linked to DAD to form a DAD/Cys layer, which effectively blocked the loaded drug DOX. The resulting OS-N=C-DAD/Cys carrier demonstrated low premature drug release, with a cumulative release of only 6.5% over 48 h under normal physiological conditions. Additionally, the carrier exhibited dual pH/GSH-responsive degradation properties. The Schiff base structure in the DAD/Cys layer enabled acid-responsive drug release, as indicated by fluorescence changes, while GSH-responsive cleavage of the disulfide bond facilitated the degradation of the Si-O-Si frameworks.

**Figure 25:**
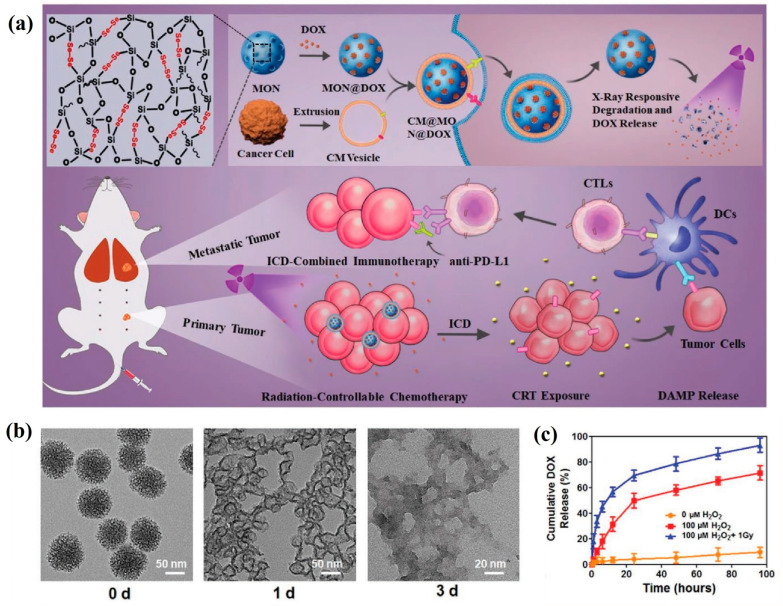
Preparation and characterization of mesoporous organosilica nanoparticles (MONs). (**a**) Schematic of synthesis of diselenide-bond-bridged MONs for low-dose X-ray radiation-controllable drug release. (**b**) TEM images of MONs showing degradation at 1 d and 3 d after 1 Gy of X-ray radiation under 100 × 10^−6^ m H_2_O_2_. (**c**) Drug release profiles in 100 × 10^−6^ m H_2_O_2_ with or without radiation. Data are presented as mean ± SD (*n* = 3). Reprinted with permission from [142]. Copyright 2020, Wiley-VCH GmbH. (MON, diselenide-bridged mesoporous organosilica nanoparticles; CM, cell membrane; ICD, immunogenic cell death; CTLs, cytotoxic T lymphocytes; DCs, dendritic cells; PD-L1, programmed death ligand 1.)

Diselenide-containing functional materials have been designed and synthesized for controlled release applications, owing to their distinctive redox-responsive properties. Shao et al. [141] introduced organosilicon segments with diselenide bonds into silica frameworks, creating biodegradable bridged organosilicon nanoparticles. These diselenide-bridged nanoparticles were capable of encapsulating cytotoxic ribonuclease A (RNase A) within their internal pores via electrostatic interactions. Furthermore, the cancer cell membrane (CM) was coated with the RNase-A-loaded diselenide-bridged MSNs to construct a bioinspired nanoplatform MSN@RNase A@CM, displaying homologous targeting and immune-evading properties. The controlled release of RNase-A occurred upon exposure to oxidative or redox conditions, triggering the complete biodegradation of the nanoparticles. In vitro and in vivo studies demonstrated their anticancer properties, including extended circulation time, enhanced tumor accumulation, and low toxicity. These findings suggest that dual-responsive and degradable organosilicon nanoparticles present a promising platform for the controlled release of biomolecules, such as proteins and nucleic acid therapeutics. The same group also developed X-ray and ROS-responsive diselenide-bridged mesoporous organosilica nanoparticles coated with 4T1 breast cancer cell membranes (CM@MON@DOX) for the controlled release of doxorubicin (DOX) at tumor sites [142]. This system demonstrated greater accumulation at tumor sites and prolonged circulation time in the bloodstream. Upon low-dose X-ray irradiation, CM@MON@DOX exhibited a degradation controlled release through the cleavage of diselenide bonds, inducing immunogenic cell death (Figure 25). The results highlighted the potential of dual-responsive diselenide-bridged organosilicon nanoparticles as a powerful tool for chemo-immunotherapy. Additionally, selenium has shown promise for coordinating metal ions or molecules. Zhang and coworkers [143] utilized diselenide-bridged mesoporous organosilicon nanoparticles (MONs) loaded with the chemotherapeutic ruthenium compound KP1339 through coordination (MON@KP1339), creating a nanoamplifier with potential for effective and safe cancer chemo-immunotherapy. The nanoplatform exhibited a controlled release pattern driven by competitive GSH-responsive coordination and carrier degradation while simultaneously inducing GSH depletion and ROS production in breast cancer cells.

Dual- or multiple-stimuli-responsive BPS carriers are generally constructed in two main ways. In one approach, a single-stimuli-responsive bridged siloxane forms the basis of the BPS material, with the surface subsequently modified by another stimuli-responsive material to create a multiple-stimuli-responsive controlled release system. Alternatively, the organic bridge groups in stimuli-responsive BPSs respond to two different stimuli, resulting in a dual-responsive system.

The development of stimuli-responsive BPS controlled release systems represents significant progress in drug delivery methods [144]. Endogenous stimulation signals, as efficient and stable triggers, can be integrated into intelligent sensing systems, enabling real-time responses to the physiological environment and the release of therapeutics with high biomedical translational value. Exogenous stimulation signals offer precise control, allowing for the regulation of signal parameters and achieving accurate timing, positioning, and dosage of drug release. Consequently, stimuli-responsive BPS systems show considerable potential for applications in disease treatment, immunobiology, diagnostics, dermatology, and cosmetology. However, challenges remain, including the poor reversibility of certain responsive groups, irreversible transformations, the limited variety of responsive groups, and low sensitivity. Therefore, future research should focus on the development of stimuli-responsive BPS biomaterials with highly controllable responsiveness, enhanced sensitivity, and improved reversibility.

## 4. Biocompatibility and Biodegradability of BPSs

The key characteristic of many degradable polymer nanoparticles lies in their exceptional biocompatibility, ensuring the absence of adverse reactions or immune rejection. Furthermore, their degradation products do not exhibit significant cytotoxicity, thus guaranteeing safety and reliability throughout the entire degradation process. These properties render these platforms highly promising in drug delivery, tissue engineering, and biomedical fields. Moghaddam and his team successfully synthesized two GSH-responsive degradable MONs by the co-condensation of TEOS with disulfide- or tetrasulfide-bridged organosiloxane monomers [145]. Experimental measurements demonstrated that the cell survival rate remained at approximately 25% even at concentrations of up to 1000 μg mL^−1^. Subsequently, they tested cell activity assays on the degradation products of these nanoparticles, which exhibited excellent biocompatibility. The in vitro cytotoxicity of tri-L-lysine-bridged BPS nanodonuts (NDs) synthesized by Maggini et al. against HeLa cells was not observed (concentrations of NDs = 20, 40, 80, and 120 μg mL^−1^) [99]. The enhanced cytotoxicity of drug-loaded nanomaterials DOX-NDs after enzymatic degradation may be attributed to the high uptake of DOX-NDs by HeLa cells, which simultaneously release their drug loads, facilitating the entry of anticancer drug into the cells. The *o*-nitrobenzyl ether-bridged polysilsesquioxane nanoparticles (LB-MSPs) have demonstrated excellent biocompatibility in HeLa cells after 12 and 24 h of incubation at concentrations of 20, 100, and 200 μg mL^−1^ [107]. Furthermore, their degradation products exposed to UV irradiation for 12 h showed no significant cytotoxicity within this concentration range and over the monitoring period. Indeed, although a wide range of biocompatible stimulus-responsive BPS materials have been extensively studied, there is a noticeable dearth of research concerning the cytotoxicity associated with the degradation products resulting from their stimuli-triggered breakdown. Consequently, this has emerged as a critical area necessitating urgent attention and comprehensive exploration by researchers.

**Figure 26:**
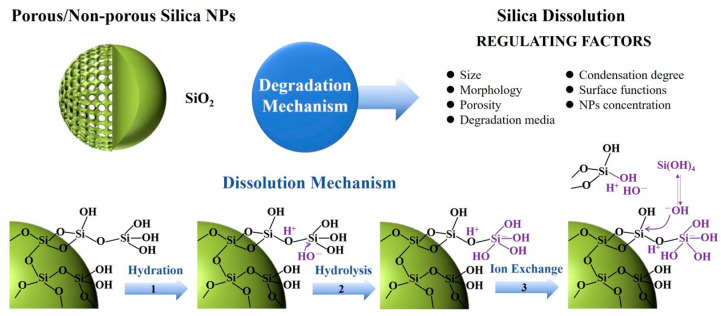
A representation of the intact and degraded structures of amorphous silica along with the mechanism and regulating factors of the degradation. Reprinted with permission from [153]. Copyright 2017, WILEY-VCH Verlag GmbH & Co. KGaA, Weinheim, Germany.

Organosilicon materials display limited biodegradability and prolonged clearance times, leading to their accumulation in organs and tissues. This accumulation poses risks of long-term toxicity and limits passive targeted specificity [146,147], creating a significant obstacle to biomedical applications and clinical translation. The degradability, toxicity, and clearance rates of organosilicon materials do not align with the timeliness standards set by the US FDA [148]. The degradation of amorphous silica in aqueous media typically occurs in three stages [149,150,151], as illustrated in Figure 26. These are (1) hydration, (2) hydrolysis, and (3) ion exchange. At the molecular level, water initially adsorbs onto the siloxane framework, promoting the hydrolysis of siloxanes into silanol. This is followed by ion exchange via nucleophilic attack by OH^-^ ions, leading to the leaching of biocompatible silicic acid and polysilicic acid, which are eventually cleared through the lymphatic system or excreted in urine. Consequently, direct interaction between silica and water molecules, as well as the framework structure, are critical determinants of biodegradation behavior. Factors affecting the biodegradation of silica dioxide include pore structure, size, shape, surface functionalization, charge, and framework composition [152,153].

The conventional silica framework consists of a -Si-O-Si- network structure. By incorporating organic components into the silica matrix, the degradation of the less condensed -Si-O-Si- network can be regulated and controlled, enhancing the biocompatibility and biodegradability of bridged polysilsesquioxanes (BPSs) [154,155]. Introducing breakable organic bridged groups into siloxane triggers degradation through stimuli-responsive reactions [156]. Organic groups responsive to stimuli, such as disulfides, tetrathiolates, sulfoxides, diselenides, Schiff bases, mannitol derivatives, amides, azo compounds, nitrophenyl groups, and coumarin groups, have been extensively reported to modulate the biodegradability of BPSs [14,147,157]. Under specific stimuli, BPSs can rapidly decompose into low-molecular-weight nanosheets, potentially alleviating metabolic issues associated with residual substances in vivo.

In the design of biodegradable and removable BPSs, factors such as size, shape, porosity, and surface characteristics must be carefully considered. Specifically, the degradation rate of BPSs can be tailored through structural modifications that disrupt organic functional groups using endogenous or exogenous stimuli [158]. Compared to traditional silica frameworks, BPS materials with organic functional frameworks exhibit faster degradation and metabolism, addressing key challenges in the clinical diagnosis and treatment of nanomaterials. Furthermore, the stimuli-triggered disassembly characteristics of organic functional bridges enable BPSs to demonstrate superior controlled release properties. Current research primarily focuses on developing stimuli-responsive organosiloxane materials and incorporating them into -Si-O-Si- frameworks to improve biodegradability. The selection of suitable organosiloxane is critical, as each stimulus source has its own advantages and limitations. Therefore, when designing BPS biomaterials for specific diseases, the most appropriate stimulus must be selected to induce degradation and facilitate the controlled release of therapeutics.

## 5. Conclusions and Future Perspective

BPSs have attracted notable attention from researchers for their biomedical applications, particularly in controlled release systems, owing to several advantages, including tunable morphological parameters (such as shape, size, and pore size), a wide range of surface functionalizations, and favorable biocompatibility. Consequently, controlled release systems based on stimuli-responsive BPSs hold substantial promise for the safe and effective treatment of diseases, leveraging stimulating factors to trigger transformations in physicochemical properties and achieve precise control over the temporal and spatial release of targeted therapeutics. Recent years have witnessed a growing focus on the design and development of novel controlled release systems utilizing stimuli-responsive BPSs, demonstrating considerable potential in targeted therapeutic delivery, controlled release, biosensing, cell imaging, biochips, and other biomedical applications.

In summary, this review discusses the design considerations, synthesis strategies, and biomedical applications of stimuli-responsive BPSs. However, most of the studies mentioned remain at the laboratory stage and require further exploration and optimization in the following areas:

(1) Biosafety issues for stimuli-responsive BPS biomaterials: Although numerous in vitro and in vivo experiments have confirmed that stimuli-responsive BPS biomaterials are generally non-toxic and non-immunogenic, factors such as material shape, size, surface charge, and functional groups may influence their toxic effects. These considerations indicate that a comprehensive biosafety evaluation of stimuli-responsive BPS biomaterials is essential. In particular, long-term toxic effects, including immunotoxicity, cardiotoxicity, nephrotoxicity, hepatotoxicity, and pulmonary toxicity, as well as biodegradation behavior and the elimination and clearance pathways of BPS systems in vivo, require thorough and systematic investigation.
(2) Scalability and reproducibility issues in manufacturing stimuli-responsive BPS materials: The scalable production of stimuli-responsive BPS materials is a critical prerequisite for their clinical application, necessitating a transition from low-yield laboratory production to ultrahigh-yield industrial processes. Maintaining consistent morphological properties of nanocarrier materials during this transition poses significant challenges. Moreover, the expected increase in manufacturing costs may hinder the industrialization of stimuli-responsive BPS materials. To address these challenges, researchers must implement effective measures across various domains, including the simplification and standardization of production processes, as well as the development of cost-effective silicon sources and coupling agents.
(3) Structural complexity of stimuli-responsive BPS materials: To address the therapeutic needs within complex pathological microenvironments, recent designs of stimuli-responsive BPS materials have increasingly focused on multifunctionality and integration for diagnosis and treatment, resulting in systems that incorporate multiple types of functional modules. However, these carriers are often “over-engineered”, leading to complex structural arrangements. An increase in the number of components within carriers corresponds to a heightened risk of therapeutic uncertainty and a diminished likelihood of clinical approval. Consequently, when designing stimuli-responsive BPS materials, a careful balance must be struck between structural complexity and functional versatility. Streamlining system design to eliminate redundant components while preserving the integrity of the desired functions is essential for establishing a simple and effective controlled release system.

The consistency of results of stimuli-responsive BPS materials in vitro and in vivo experiments also requires further exploration. The extensive literature supports the performance of stimuli-responsive BPS materials in in vitro experiments, particularly regarding on-demand release capabilities and targeted delivery under specific stimuli. However, the presence of multiple physiological barriers, such as blood, tissue, and cellular barriers, alongside complex pathological environments in living organisms, can impede the specific accumulation and controlled release of stimuli-responsive carriers at targeted tissues, often leading to negative outcomes in in vivo experiments. Therefore, researchers must develop organosilicon-based nano-release systems that possess “stealth” functions (i.e., the ability to evade clearance by the immune system) and targeted tissue-enriched permeation capabilities (i.e., specific accumulation and efficient diffusion within diseased tissues). Strategies such as biocompatible polymer coatings (e.g., polyethylene glycol), biomimetic camouflage (e.g., cell membrane embedding), and targeted ligand modification (e.g., RGD peptide) should be employed to enhance these characteristics. Additionally, the lack of clinical trials investigating whether the biological behavior of organosilicon materials in humans concerning distribution, accumulation, degradation, and metabolism mirrors that observed in model animals remains a significant concern that warrants continued attention and exploration by researchers.

Conventional stimulation conditions, singular stimulation modes, and simplistic functional outputs are insufficient to satisfy the demands of the biomedical industry. The development of innovative stimulation conditions and functional responses that exhibit synergistic and logical control has emerged as a challenging yet crucial focus in constructing stimuli-responsive controlled release systems. Enhancing the system’s synergistic control capabilities can diversify the functionalities of stimuli-responsive systems, making them more practical for the development of intelligent devices or nano-functional entities based on BPS biomaterials. This advancement may pave the way for the rational use of organosilicon materials and the creation of new materials. Overall, this review can serve as a reference for applying stimuli-responsive BPS biomaterials in biomedical fields while inspiring future development.

## Figures and Tables

**Table 1:** Summary of reported redox-responsive BPS platforms for controlled release systems.

Matrix	Particle Size	Pore Size	Sensitive Bonds	Trigger	Targeted Therapeutics	Application	Ref.
DIS2 and DISP BS NPs	40, and 50 nm	—	Disulfide	GSH	—	Two-photon-excited imaging and therapy of breast cancer cells	[49]
PROMON-CaC	65 nm	—	Disulfide	GSH	Prochloraz	Sustainable plant disease management and precision farming	[50]
DSMSNs@Res@CS	233.3 nm	~50 nm	Tetrasulfide	GSH	Resveratrol	Oral delivery platform	[51]
PRO@DMON–GA–Fe(III) NPs	80 nm	—	Disulfide	Reducing environments generated by the fungus	Prochloraz	Plant disease management	[52]
NaCl@ssss-VHMS	~150 nm	~3.6 nm	Tetrasulfide	GSH	Na^+^/Cl^−^	Cancer therapeutic agent	[53]
DSMSNs@Res@HA	237.73 nm	~50 nm	Tetrasulfide	GSH	Resveratrol	Attenuate acrylamide-induced toxicity	[54]
UPOMs	103 nm	—	Disulfide	GSH	Chlorin e6 and DOX	Combination therapy of chemotherapy and NIR-mediated PDT	[55]
UMONs–LA–Au	sub-50 nm	2.4 nm	Disulfide	GSH	L-Arginine	Tumor-specific precision cascaded therapy	[56]
avermectin@MSNs-ss-starch	80.3 ± 8.7 nm	—	Disulfide	GSH	Avermectin	Targeted pesticide delivery	[57]
Paclitaxel/IR820@ HMONs-PEG	125.3 ± 9.15 nm	5.2 nm	Disulfide	GSH	Paclitaxel and photothermal agent	Photothermal-enhanced chemotherapy of tumor	[58]
Mn_3_O_4_@PDOMs-GOD	~177 nm	—	Disulfide	GSH	Mn_3_O_4_ and glucose oxidase	Starvation and chemodynamic therapy	[59]
MON-TPGS-DOX	70 nm	—	Tetrasulfide	GSH	DOX	H_2_S-enhanced tumor chemotherapy	[60]
Ag-MONs@GEN	200 nm	1.68 nm	Disulfide	GSH	Gentamicin and nanosilver	Synergistic treatment of antibiotic-resistant bacteria	[61]
CHX@MONs	812 ± 27 nm	3.5 nm	Disulfide	GSH	Chlorhexidine	Treatment of bacterial infections	[62]
(CisPt+EA)@SHMONs	70 nm	3.58 nm	Disulfide	GSH	Cisplatin and ethacrynic acid	Chemotherapy against drug-resistant cancers	[63]
Apt-RBC-HMOS@DOX	295 ± 1.3 nm	1.2 nm	Tetrasulfide	GSH	DOX	Cancer therapy	[64]
DTX@IPOMs	20 nm	—	Disulfide	GSH	Docetaxel	Ultrahigh dosage chemotherapy	[65]
DTeMSN@PEG-CCM	~40 nm	7.6 nm	Ditelluride	ROS and GSH	DOX	Fluorescence-guided drug delivery	[32]
MON-Pt@CM	60 ± 5 nm	6.2 nm	Diselenide	GSH	Pt	Pt-based chemotherapy	[66]
^Se^MSNs@CS@Ap	185.1 nm	—	Diselenide	ROS	Cromoglycate sodium	Clinical generalization of allergic diseases	[67]
DOX@HMONs@PDA-mPEG	130.7 ± 4.3 nm	11.1 nm	Thioacetal	ROS	DOX	Drug delivery	[68]
T-BS-NPs@M	58.5 nm	—	Thioketal	ROS	Metformin	Combinatorial therapy of metformin and fasting therapy	[69]

**Table 2:** Summary of reported pH-responsive BPS platforms for controlled release systems.

Matrix	Particle Size	Pore Size	Sensitive Bonds	Trigger	Targeted Therapeutics	Application	Ref.
M1	100 nm	—	Triazine derivative	pH	Cyanuric acid	Delivery system	[84]
BS	—	—	Triazine derivative	pH	5-fluorouracil	Controlled drug release	[85]
Nano-BS	~300 nm	—	Triazine derivative	pH	Cyanuric acid	Combination of chemotherapy and fluorescence imaging	[86]
HMCs	100 nm–3.5 μm	—	Diurea-functionalized pyridine	pH	5-fluorouracil and ibuprofen	Delivery system	[87]
His-PMO	—	7.8 nm	Histidine	pH	Paclitaxel	Rapid accumulation of drugs	[88]
S–MON	57.8 nm	2.77 nm	Benzoic–imine bonds	pH	DOX	Cancer therapeutics	[89]
PS@SiO_2_*	118 ± 8 nm	—	Diimine	pH	—	Drug delivery	[90]
PBHMONs	~100 nm	2.2 nm	Acetal moieties	pH	DOX	Efficient anticancer drug delivery	[91]
ICPTES–sorbitol SNPs	340 ± 29 nm	—	Carbamate linkages	pH	—	Oral-based drug delivery	[92]

**Table 3:** Summary of reported enzyme-responsive BPS platforms for controlled release systems.

Matrix	Particle Size	Pore Size	Sensitive Bonds	Trigger	Targeted Therapeutics	Application	Ref.
S2	100–150 nm	—	Azobenzene linkers	Azoreductase	Ibuprofen	Colon-specific drug delivery	[95]
AZO-B and AZO-E	—	1.6 nm (AZO-B), 2.7 nm (AZO-E)	Azobenzene linkers	Azoreductase	DOX	On-demand drug delivery	[96]
BS NPs	295 nm	—	Oxamide	Trypsin	—	Imaging nanoprobes	[97]
MON	181 ± 23 nm	2.1 nm	Oxamide	Trypsin	DOX	Disease-targeted treatments	[98]
NDs	220 ± 36 nm	—	Tri-L-lysine	Peptidase	DOX	Drug delivery	[99]

**Table 4:** Summary of reported exogenous stimuli-responsive BPS platforms for controlled release systems.

Matrix	Particle Size	Pore Size	Sensitive Bonds	Trigger	Targeted Therapeutics	Application	Ref.
BS NPs	100–200 nm	—	*o*-Nitrophenylene-ammonium	UV light	Plasmid DNA	On-demand delivery	[102]
CBPS	—	—	Diethylaminocoumarin-4-yl	UV light	Protein	Protein micropatterning	[103]
NBPS	124 ± 12 nm	—	*o*-Nitrobenzyl	UV light	DOX	Precisely controlled drug release	[104,105]
HMONs@GOQDs	—	~3.94 nm	9, 10-Dialkoxyanthracene	UV light	DOX	Photocontrolled drug release	[106]
LB-MSPs	409 ± 80 nm	2.4 nm	*o*-Nitrobenzyl ether	UV light	7-Dehydrocholesterol	Quantitatively drug release	[107]
Se-MSN-PEG@M&D	72.5 ± 4.8 nm	6.35 nm	Diselenide	NIR and ROS	DOX	Cascade-amplifying chemo-photodynamic therapy	[108]
ID@M-N	115 nm	2.69 nm	Diselenide	NIR and ROS	DOX	Chemo-immunotherapy	[109]
HMONs@CuS/DOX@PCM	less than 200 nm	2.7 nm	Disulfide	NIR and GSH	DOX	Drug delivery and synergistic chemo and thermal therapy	[110]
Dox@CuS-BMSN-HA	37.11 ± 6.59 nm	—	Tetrasulfide	NIR and GSH	DOX	Chemo-photothermal synergistic therapy	[111]
CM@M-MON@Ce6	—	3.8 nm	Disulfide	Magnetic and GSH	—	PDT and magnetic hyperthermia synergistic anticancer	[112]
HMONs-MnPpIX-PEG	—	3.4 nm	Disulfide	US and ROS	—	Ultrasound therapy	[113]

**Table 5:** Summary of reported multiple-stimuli-responsive BPS platforms for controlled release systems.

Matrix	Particle Size	Pore Size	Sensitive Bonds	Trigger	Targeted Therapeutics	Application	Ref.
HMOPM	106.1 ± 11.1 nm	~3.7 nm	Tetrasulfide	pH and GSH	Mn_2_(CO)_10_	Tumor-specific self-assembly and synergistic cancer therapy	[134]
CuS@BSA-HMONs-DOX	117.6 nm	1.78 nm	Disulfide	GSH, pH, and NIR	DOX	Photoacoustic imaging guided chemo-photothermal therapy	[135]
DOX-PCMONSs	320 nm	4 nm	Disulfide	GSH, pH, and NIR	DOX	Theranostic nanoplatform	[136]
IR&DOX@NC	—	—	Disulfide	Enzyme, GSH, and NIR	DOX and IR820	Multimodal cancer therapy	[137]
YSPMOs(DOX)@CuS	222.6 nm	2.67 nm	Disulfide	GSH, pH, and NIR	DOX	Chemo-photothermal synergistic therapy	[138]
ZDOS NPs	158 nm	0.6 nm	Disulfide	pH and GSH	DOX	Controlled release and cancer treatment	[139]
OS-N=C-DAD/Cys	192 nm	11.76 nm	Disulfide	pH and GSH	DOX	Monitor drug release	[140]
MSN@RNase A@CM	50 nm	8.5 nm	Diselenide	GSH and ROS	RNase A	Biomacromolecule delivery	[141]
CM@MON@DOX	60 nm	4.2 nm	Diselenide	ROS and X-ray	DOX	Breast cancer chemo-immunotherapy	[142]
MONs@KP1339	65 nm	6.4 nm	Diselenide	GSH and coordination	Ruthenium compound	Breast cancer chemo-immunotherapy	[143]

## Data Availability

No new data were created or analyzed in this study. Data sharing is not applicable to this article.

## Abbreviations

BPS, bridged polysilsesquioxane; BPNPs, BPS nanoparticles; MONs, mesoporous organosilicon nanoparticles; MSN, mesoporous silica nanoparticle; HMONs, hollow organosilicon nanoparticles; PMO, periodic mesoporous organosilicon; CTAC, cetyltrimethylammonium chloride; CTAB, cetyltrimethylammonium bromide; TEOS, tetraethoxysilane; TMOS, tetramethoxysilane; GSH, glutathione; ROS, reactive oxygen species; DOX, doxorubicin; DTT, dithiothreitol; CaC, calcium carbonate; PRO, prochloraz; Res, resveratrol; CS, chondroitin sulfate; Pt, platinum; PEG, poly(ethylene glycol); HMCs, mesoporous organosilica hybrid microcarriers; 5-FU, 5-fluorouracil; IBU, ibuprofen; PTX, paclitaxel; PS, polystyrene; ^1^O_2_, singlet oxygen; GOQD, graphene oxide quantum dot; PTT, photothermal therapy; PDT, photodynamic therapy; UV, ultraviolet; NIR, near-infrared; NIPAM, N-isopropylacrylamide; ICG, indocyanine green; PCMs, phase-change materials; Ce6, chlorin e6; US, ultrasound; HIFU, high-intensity focused ultrasound; SDT, sonodynamic therapy; BTEB, bis(triethoxysilyl)phenylene; BTES, bis [3-(triethoxysilyl)propyl]tetrasulfide; DAD, dialdehyde dextrin; Cys, cystamine.
